# Genome-wide identification of resistance genes and cellular analysis of key gene knockout strain under 5-hydroxymethylfurfural stress in *Saccharomyces cerevisiae*

**DOI:** 10.1186/s12866-023-03095-2

**Published:** 2023-12-04

**Authors:** Qian Li, Peng Feng, Hao Tang, Fujia Lu, Borui Mou, Lan Zhao, Nan Li, Yaojun Yang, Chun Fu, Wencong Long, Ximeng Xiao, Chaohao Li, Wei Wu, Gang Wang, Beidong Liu, Tianle Tang, Menggen Ma, Hanyu Wang

**Affiliations:** 1 College of Life Science, Leshan Normal University, No. 778 Binhe Road, Leshan, 614000 Sichuan China; 2 Department of Applied Microbiology, College of Resources, Sichuan Agricultural University, No. 211 Huimin Road, Wenjiang, Chengdu, Sichuan 611130 People’s Republic of China; 3 Bamboo Diseases and Pests Control and Resources Development Key Laboratory of Sichuan Province, College of Life Science, Leshan Normal University, Leshan, 614000 Sichuan China; 4 Key Laboratory of Land Resources Evaluation and Monitoring in Southwest, Ministry of Education, Sichuan Normal University, Chengdu, 610068 China; 5 Jiangxi Forestry Science and Technology Promotion and Publicity Education Center, Nanchang, 330000 Jiangxi China; 6 Leshan Institute of Product Quality Supervision and Testing, Leshan, 614000 Sichuan China; 7 Engineering Research Center of Sichuan Province Higher School of Local Chicken Breeds Industrialization in Southern Sichuan, College of Life Science, Leshan Normal University, Leshan, 614000 Sichuan China; 8 Department of Chemistry and Molecular Biology, University of Gothenburg, Medicinaregatan 9C, 413 90 Göteburg, Sweden; 9 State Key Laboratory of Subtropical Silviculture, School of Forestry and Biotechnology, Zhejiang A&F University, Lin’an, Hangzhou, 311300 Zhejiang China; 10 Key Laboratory of Tropical Transitional Medicine of Ministry of Education, Hainan Medical University, No.3 Xueyuan Road, Haikou, 571199 Hainan China

**Keywords:** *Saccharomyces cerevisiae*, *SIW14*, 5-Hydroxymethylfurfural, Tolerance

## Abstract

In bioethanol production, the main by-product, 5-hydroxymethylfurfural (HMF), significantly hinders microbial fermentation. Therefore, it is crucial to explore genes related to HMF tolerance in *Saccharomyces cerevisiae* for enhancing the tolerance of ethanol fermentation strains. A comprehensive analysis was conducted using genome-wide deletion library scanning and SGAtools, resulting in the identification of 294 genes associated with HMF tolerance in *S. cerevisiae*. Further KEGG and GO enrichment analysis revealed the involvement of genes *OCA1* and *SIW14* in the protein phosphorylation pathway, underscoring their role in HMF tolerance. Spot test validation and subcellular structure observation demonstrated that, following a 3-h treatment with 60 mM HMF, the *SIW14* gene knockout strain exhibited a 12.68% increase in cells with abnormal endoplasmic reticulum (ER) and a 22.41% increase in the accumulation of reactive oxygen species compared to the BY4741 strain. These findings indicate that the *SIW14* gene contributes to the protection of the ER structure within the cell and facilitates the clearance of reactive oxygen species, thereby confirming its significance as a key gene for HMF tolerance in *S. cerevisiae*.

**Supplementary Information:**

The online version contains supplementary material available at 10.1186/s12866-023-03095-2.

## Background

With the depletion of oil resources and growing concern over environmental problems, biomass resources are now the green channel to realize the transition of oil resources (a likely substitute for traditional fossil fuels) [1]. The first-generation biomass fuel is generated from sugar, starch, vegetable oil and other raw materials, whose mass production will exert pressure on the food supply. The second generation of biomass fuel is produced from main raw materials such as wood waste, crop residues, specific energy crops and other lignocellulose [2], bringing about a reduction in the crop consumption and a brighter outlook for its development. Lignocellulosic biomass is the most abundant and sustainable resource on the earth so it has been widely used to produce biofuels, biochemical reagents and food additives through microbial fermentation [3–5]. However, lignocellulosic biomass needs to be pre-treated to produce monosaccharides for microbial fermentation. Among various pretreatment methods, although dilute acid hydrolysis is the simplest, fastest and most cost-saving method., it is inevitably accompanied by the production of inhibitors, which seriously retard the growth and fermentation of subsequent microbial cells [6]. 5-hydroxymethylfurfural (HMF) is one of the most representative furan inhibitors in lignocellulosic hydrolysates. There have been substantial efforts devoted to study on the tolerance mechanism of microorganisms to HMF in terms of cell morphology, oxidative stress, protein response, gene response and REDOX balance where HMF was found not only to change the morphology of cells and organelles but also cause accumulation of intracellular reactive oxygen species, which induced significant changes in cells from gene expression to transcription to protein translation as well as disruption of intracellular redox homeostasis [7, 8]. Microbial tolerance to HMF is related to the restoration or adjustment of intracellular REDOX balance. The faster the microorganisms can recover or readjust the REDOX balance to the presence of HMF, the more tolerant the microorganisms to it will be [6]. And some studies have found that microorganisms can detoxify HMF by degrading it into low-toxic substances. For example, under aerobic culture conditions, *Bacillus pasteuri*, *Bacillus megaterium*, *Bacillus cereus*, *Bacillus subtilis*, and *Bacillus SPP*. can convert HMF into low-toxicity 2-furanoic acid and 5-hydroxymethyl-2-furanoic acid (HMFCA) to varying degrees [9]. However, the current research on HMF tolerance mechanisms is not sufficient, so the specific mechanisms need to be further explored.

*S. cerevisiae*, the simplest model eukaryote, had its genome sequenced 20 years ago [10]. Based on its genome sequence, scientists have annotated 5,907 potential protein-coding genes and 425 potential RNA genes. Most of these genes or Open Reading frame (ORFs) protein functions have been confirmed [11]. It can be said that *S. cerevisiae* is the best classic model organism to study genetic interactions. To study the function of genes, scientists constructed a library of non-essential genes (about 4,400) knock-out [12], a temperature-sensitive library of essential genes (about 1,000) [13], a library of overexpression of genes (more than 5,000) [14], and a library of green fluorescent tags of genes (more than 5,000) of *S. cerevisiae* [15]. Based on *S. cerevisiae* library screening technology, key genes in the library can be identified by visual phenotyping. Gorsich et al. treated the knock-out library of non-essential genes in *S. cerevisiae* with furfural, and screened out 62 genes related to furfural tolerance [16]. In order to systematically study the functional interactions between the genes, Boone et al. developed synthetic gene array analysis (SGA) by crossing yeast single mutant strains with gene knock-out library, which can automatically isolate yeast double mutants [17]. The interaction between genes is determined by the death and slow growth of double mutants, facilitating large-scale mapping of genetic interactions. Hill et al. isolated Metacaspase, a caspase homologue, using the SGA technique and demonstrated that Metacaspase is associated with lifespan control [18]. Hanzen et al. found that REDOX protein Tsa1 screened by SGA technology can recruit molecular partners to misfolded proteins to achieve lifespan control [19]. Studies on the mechanism of 5-hydroxymethylfurfural tolerance using SGA technology have not been reported previously.

Therefore, this experiment exposed *S. cerevisiae* whole-gene knockout strains (about 4000 strains) to HMF, a representative inhibitor of furans, dissected the effect of inhibitor on the growth of each strain utilizing SGAtools, and completed the statistical analysis of tolerance and sensitivity of knockout strains. Our study screened out the key genes related to HMF tolerance and selected some for verification. These efforts are likely to serve as reference and guidance for the transformation of highly tolerant *S. cerevisiae* strains.

## Materials and methods

The non-essential gene knock-out strain bank of *S. cerevisiae* used in this experiment was donated by Professor Beidong Liu of University of Gothenburg, Sweden [20]. 5-hydroxymethylfurfural (abbreviation: HMF) was purchased from Sigma, yeast extraction powder, Geneticin (G418) from Thermo Scientific, DMSO from Solarbio, agar, peptone, glucose, ethanol, sodium chloride and other reagents were purchased from Chengdu Vanke Co., LTD.

Among all the fluorescent staining agents used in the study, 2′7'-dichlorofluorescein diacetate was purchased from Sigma, Diaminophenylindole (DAPI) from Sangon Biotech (Shanghai) Co., Ltd, Mito Tracker Green FM, Yeast Vacuole Membrane Marker MDY-64 and ER-Tracker Red dye from Thermo Scientific.

### HMF concentration screening

To prepare the YPD liquid medium, combine 1% yeast extract powder, 2% peptone, and 2% glucose. However, for the solid medium, an additional 2% agar was added. YPD + G418 medium: Add G418 (final concentration 100 mg/L) to YPD medium. Before the spot test, streak strains (knock out strains) on YPD + G418 plate and obtain single colonies after culture, which were inoculated into 30 mL YPD + G418 liquid medium in a 100 mL triangular bottles. After being cultured at 30 ℃ on a lab rotary shaker at 200 r/min for 18–24 h, sample or cell or Cell Cultures were obtained. And used to conduct spot test experiment where the growth curve of the liquid culture was determined after observation and photo-taking.

In the spot test, the cell density concentration (OD_600_) of the yeast culture above was uniformly calibrated to 0.5, and then a tenfold serial dilution was carried out to obtain a series of yeast solution of decreasing concentrations. 5 uL of diluted yeast solution was pipetted onto YPD + G418 solid medium containing different concentrations of HMF using a cascade pipette. After 3 to 4 days of culture, observation and photography were taken.

In the liquid culture experiment, the cell density concentration (OD_600_) of the cultured yeast solution above was uniformly calibrated as 0.25 and added into YPD + G418 liquid medium containing different concentrations of HMF. Spectrophotometer was used to measure the cell concentration at 6 h, 12 h, 18 h, 24 h, 36 h and 48 h, respectively and generated a growth curve.

### Screening of HMF tolerant or sensitive knockout strains

Knockout strains with genes related to HMF tolerance were selected from the non-essential gene knockout strain bank (about 4,000 strains), and arranged in 384-format on 384-well cell culture plates. Next, the strains from the 384-well frozen cutting board were copied onto the YPD + G418 ager plate using a 384-pinning Singer rotor. Four copies of the knockout strains were grown on the YPD + G418 ager plate. After incubation, the strains on the plate were replicated in a new YPD + G418 plate containing 60 mM HMF. Incubating for two days, we took high-definition pictures of the tablet and used SGAtools (http://sgatools.ccbr.utoronto.ca/) to evaluate the growth of individual strains with HMF treatment [21]. The score for each strain was calculated based on standard strain BY4741 colony size for control. According to the growth status, HMF-tolerant (The colony was smaller than the control) or sensitive (The colony was larger than the control) knockout strains were screened, and knockout genes with score ≥ 0.2 or ≤ -0.2 obtained from the HMF tolerance or sensitivity screening were selected as candidate tolerance or sensitivity genes. The score screening criteria have refered to previous studies [20]. When the score value was ≥ 0.2, the corresponding knockout gene of the knockout strain is a sensitive gene, and the strain will be more tolerant to HMF after the knockout of this type of gene. If the score value is ≤ -0.2, the corresponding knockout gene of the knockout strains is a tolerance gene, after the knockout of this type of gene, the strain will be less tolerant to HMF. Finally, KEGG and GO enrichment analysis were conducted on the above candidate genes using Cytoscape software [22].

### Verification of HMF tolerant or sensitive gene knockout strains

Activated yeast solution of the original strain BY4741 and the gene knockout strain were spotted onto YPD + G418 ager plate containing 60 mM HMF and no HMF at certain concentrations, respectively and their growth were monitored and their photos were taken for comparative analysis.

For observation and verification of subcellular structure, the original strain BY4741 (CK) and the candidate gene knockout strain were first cultured 20 h (Yeast cells in a stable state, Figure S1) at 30 ℃ and 200 r/min in YPD + G418 liquid medium (cells of 0 h were collected here). The original strain BY4741 and the gene knockout strain which had been cultured overnight were incubated in YPD + G418 liquid medium containing 60 mM HMF (OD_600_ = 0.8), followed by cultivation at 30 ℃ and 200 r/min for 3 h (cells of 3 h were collected here). The 0 h and 3 h yeast solution were inserted into 1.5 mL centrifugal tube (OD_600_ = 1.0), respectively. The dyes 2′7’-DCFdiacetate, Mito Tracker Green FM, Vacuole Membrane Marker MDY-64, diamino phenylindole (DAPI), and ER-Tracker Red were added into centrifuged yeast solution (the dyes were thawed in advance), and subcellular observation of Reactive Oxygen Species (ROS) accumulation, chromatin disturbance, mitochondrial membrane damage, endoplasmic reticulum damage and vacuolar membrane damage were conducted using a fluorescence microscope equipped with DIC, GFP, Rhod and DAPI filters, and the percentage of their different morphologies were calculated [23–25].

## Result

### Inhibitor concentration screening

**Fig. 1:**
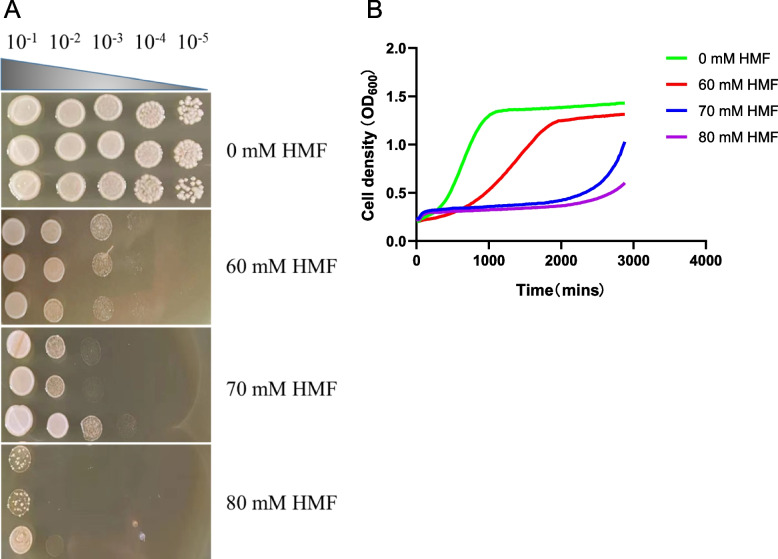
Spot test (**A**) and growth curve (**B**) of BY4741 at different HMF concentrations

In this experiment, HMF, the representative inhibitor of furans, was selected to screen out genes related to sensitivity and tolerance to HMF, a byproduct of lignocellulosic hydrolysis. Since high throughput screening was used to identify inhibitor tolerant or sensitive knockout strains, the optimal inhibitor concentration must be used. In the spot test assay, the optimal concentration of inhibitors was *S. cerevisiae* with semi-inhibition, that is, the number of recovered colonies in the medium containing inhibitors was only half of that in the medium without inhibitors. The optimal inhibitory concentration in liquid medium makes the growth retardation period twice as long as that without inhibitor. Therefore, four HMF concentration gradients were set with reference to previous studies [26]., and the growth of strain BY4741 at these concentrations was measured to determine the optimal concentration. The number of recovered colonies at a concentration of 60 mM HMF was half that at a concentration of 0 mM HMF, and only approximately one-fourth of the colonies resumed growth when concentration was increased to 70 mM and 80 mM (Fig. 1A). In YPD liquid medium containing different concentrations of HMF, the doubling time is extended 10 h at the concentration of 60 mM HMF whereas when exposed to HMF at 70 mM and 80 mM the cells were so severely inhibited that they didn’t start growing until 2 days later (Fig. 1B). Accordingly, 60 mM HMF was selected as the optimal screening concentration for non-essential gene knockout library in this study.

### Whole gene knockout library screening of HMF

**Fig. 2:**
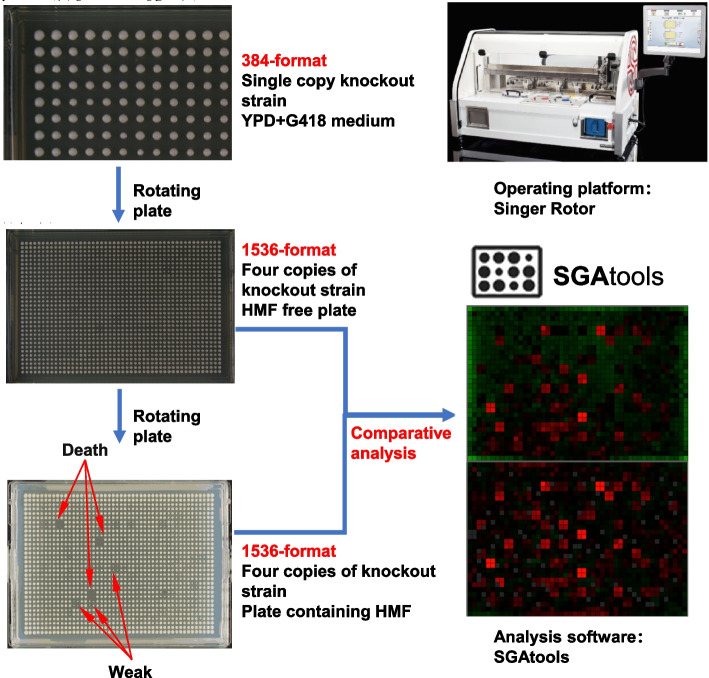
Technical route of sensitivity analysis of whole-genome knockout strains under HMF

Singer Rotor was used to replicate about 4000 *S. cerevisiae* gene knockout strains for 4 replicates in 384-format, resulting in 1536-format. The strains in the 1536-format were incubated on culture plates containing HMF at 30 ℃ for 48 h and then photographed (Fig. 2). The comparison of control and experimental plates showed that the inhibitors caused weakness or lethality (i.e., sensitivity) in knockout strains, and the corresponding genes of these strains were tolerance-related candidate genes. Taking SGA-V2-2 culture plates as an example, *Rvs161Δ* (*RVS161* knockout strain) and *Pat1Δ* (*PAT1* knockout strain) were significantly inhibited in the 30 mM FF treatment group compared with the control group (Fig. 2). By SGAtool analysis, the score of *Rvs161Δ* and *Pat1Δ* strains were -0.93 and -0.44, respectively. The colony size of these sensitive deletion mutants was significantly smaller than that of the control group, indicating that 60 mM HMF had a significant inhibitory effect on them. Screening under other inhibitor conditions was performed as above. Finally, screening was completed for fourteen 1536-format colonies (about 4,000 gene deletion strains) on YPD + G418 agar plates containing 60 mM HMF. After the images of the control and experimental plates were collected, they were uploaded to the SGAtools website for quantification analysis (Fig. 2). Screening score set to ≥ 0.2 or ≤ -0.2, 202 candidate sensitive knockout strains and 92 candidate tolerant knockout strains were found with the presence of inhibitors HMF at 60 mM (Table S1).

**Fig. 3:**
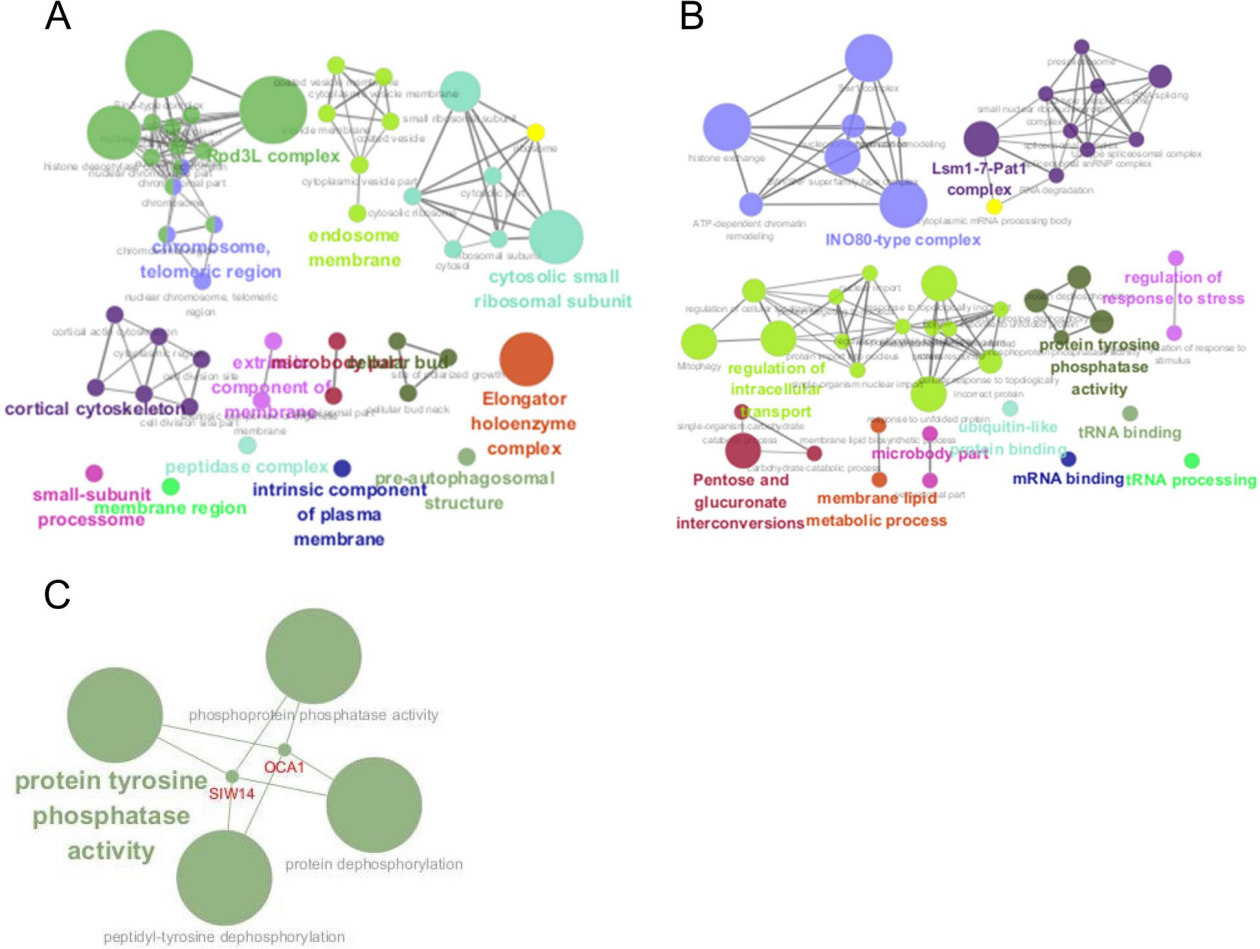
Enrichment map of KEGG and GO genes for tolerance or sensitivity to HMF in *S. cerevisiae species*. (**A**) Enrichment of related genes sensitive to HMF in S. cerevisiae BY4741. (**B**) Enrichment of HMF-tolerant genes in *S. cerevisiae* BY4741. (**C**) Protein phosphorylation modification pathway in *S. cerevisiae* BY4741 shows tolerance to HMF related genes

Then, KEGG and GO enrichment analysis were carried out to analyze the sensitive and tolerant genes using Cytoscape software [22]. The sensitive genes were mainly concentrated in cytoplasmic sac, peroxidase tissue and S-adenosine methionine-dependent methyltransferase pathways (Fig. 3A). Tolerance-related genes are mainly concentrated in membrane fluidity, IN0-80 type perfection, intracellular transport regulation and protein phosphorylation (Fig. 3B). Due to the large number of related pathways, this study mainly focus on related genes in protein phosphorylation modification pathways. As illustrated in Table 1, there are two tolerance genes enriched in the protein phosphorylation pathway: *OCA1* and *SIW14*, with screening score of -0.31 and -0.42, respectively. Both *OCA1* and *SIW14* genes are associated with protein tyrosine phosphatase activity, phosphoprotein phosphatase activity, peptidyl tyrosine dephosphorylation, and protein dephosphorylation (Fig. 3C) [22].

**Table 1:** Key genes related to HMF tolerance by protein phosphorylation pathway

AN	AO	NC size (EX)	NC std. (EX)	NC size (CN)	NC std. (CN)	Score	Score stdev	*p*-Value
*OCA1*	*YNL099C*	0.78	0.01	1.09	0.03	-0.31	0.01	0.00
*SIW14*	*YNL032W*	0.72	0.02	1.12	0.12	-0.42	0.02	0.00

Note (Note): *AO* Array ORF, *AN* Array Name, *NC size* Normalized colony size, *NC std*. Normalized colony std. dev., *EX* Experiment, *CN* Control

### Tolerance gene verification experiment

**Fig. 4:**
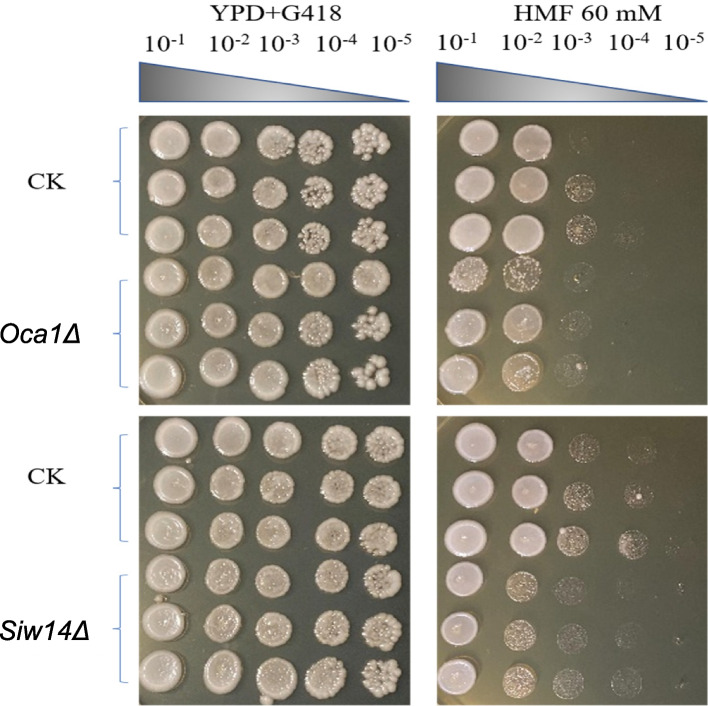
Spot test verification

Two genes, *OCA1* and *SIW14*, were enriched by KEGG and GO in the protein phosphorylation modification pathway. By observing BY4741 standard strain (CK), *Oca1Δ* (*OCA1* knockout strain) and *Siw14Δ* (*SIW14* knockout strain) through spot test. It was found that the performance of *Siw14Δ* showed the greatest difference from that of the original strain BY4741. BY4741 (CK), *Oca1Δ* and *Siw14Δ* were diluted at a certain concentration gradient, and were then spotted on solid medium YPD + G418 without HMF and YPD + G418 containing 60 mM HMF, respectively (Fig. 4). Each strain underwent three replicates, and a tenfold serial dilution was performed to establish five distinct cell concentrations for each strain. When cultivated on YPD + G418 solid medium, all three strains exhibited similar growth, suggesting no significant differences among their growth (Fig. 4). When cultured simultaneously in YPD + G418 containing 60 mM HMF, both BY4741 and *Oca1Δ* exhibited a similar pattern, with approximately two-fifths of their colonies resuming growth, indicating no significant difference between the two strains (Fig. 4). In contrast, *Siw14Δ* exhibited significantly fewer colonies recovering growth compared to BY4741 (Fig. 4). It was obvious that *Siw14Δ* is less tolerant to HMF, so further validation of *SIW14* gene will follow.

### Reactive Oxygen species

**Fig. 5:**
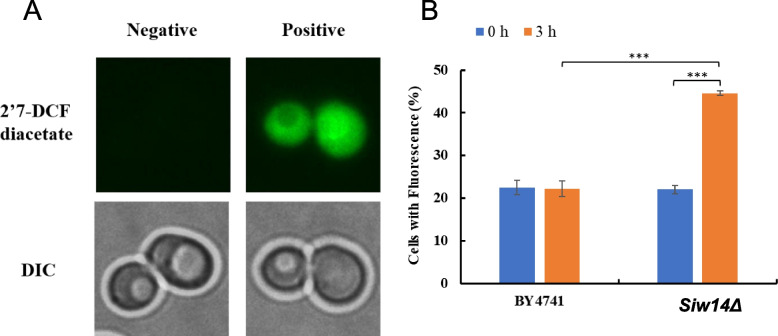
Reactive oxygen species accumulation in *S. cerevisiae*. (**A**) Accumulation of reactive oxygen species in cells. (**B**) The proportion of cells at 0 mM HMF that contained reactive oxygen species after treatment for 0 and 3 h. 2′7'-DCF diacetate (top column): reactive oxygen species indicator dye. DIC (down column): differential interference microscope. Negative: no signal; Positive: there is a signal. *** *p* < 0.001 indicates significant differences. The data represent aver-ages of three experiments. At least 100 cells were examined on each bright-field image(Figure S2, S3)

There are two different morphologies presented with and without reactive oxygen species in the cells: when reactive oxygen species accumulated in the cells, cells stained with 2′7'-DCF diacetate showed fluorescence signals of reactive oxygen species (Fig. 5A). Cells containing reactive oxygen were 22.48% and 22.26%, respectively, after 0 h and 3 h HMF treatment, the unnoticeable gap indicating that BY4741 standard strain (CK) was able to fully metabolize reactive oxygen (Fig. 5B). However, 22% and 44.67% of cells with *Siw14Δ* (*SIW14* knockout strain) were positive for reactive oxygen species at 0 h and 3 h, respectively (Fig. 5B), indicating that the knockout of *SIW14* gene impaired the ability of the strain to metabolize reactive oxygen species and caused its accumulation in cells.

### Mitochondria

**Fig. 6:**
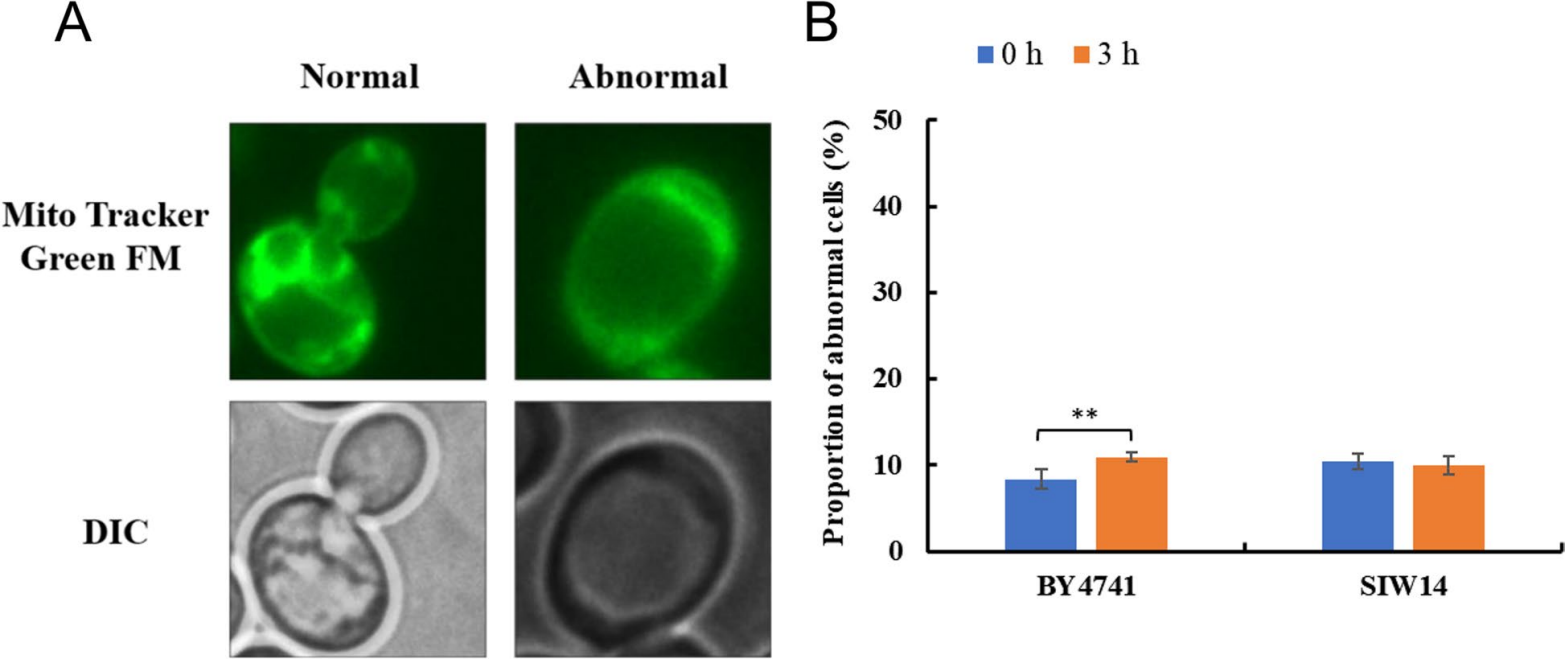
Mitochondrial morphological changes in *S. cerevisiae*. (**A**) Different morphology of mitochondria in cells. (**B**) The proportion of cells at 0 mM and 60 mM HMF that displayed abnormal mitochondria after treatment for 0 and 3 h. Mito Tracker Green FM: the mitochondria-specific dye. DIC: differential interference microscope. ** *p* < 0.01 indicates significant differences. The data represent averages of three experiments. At least 100 cells were examined on each bright-field image(Figure S2, S3)

Two different morphologies of mitochondria were observed under HMF stress by fluorescence microscopy (Fig. 6A). The percentage of cells with mitochondrial damage at 0 h and 3 h of standard strain BY4741 (CK) stood at 8.39% and 10.95% (slightly higher), respectively (Fig. 6B). The percentage of cells with mitochondrial damage at 0 h and 3 h of *Siw14Δ* (*SIW14* knockout strain) was 10.43% and10%, respectively (Fig. 6B). In conclusion, HMF slightly damages the mitochondrial structure of standard strain BY4741, but does not affect the mitochondrial structure of *Siw14Δ.*

### Endoplasmic reticulum

**Fig. 7:**
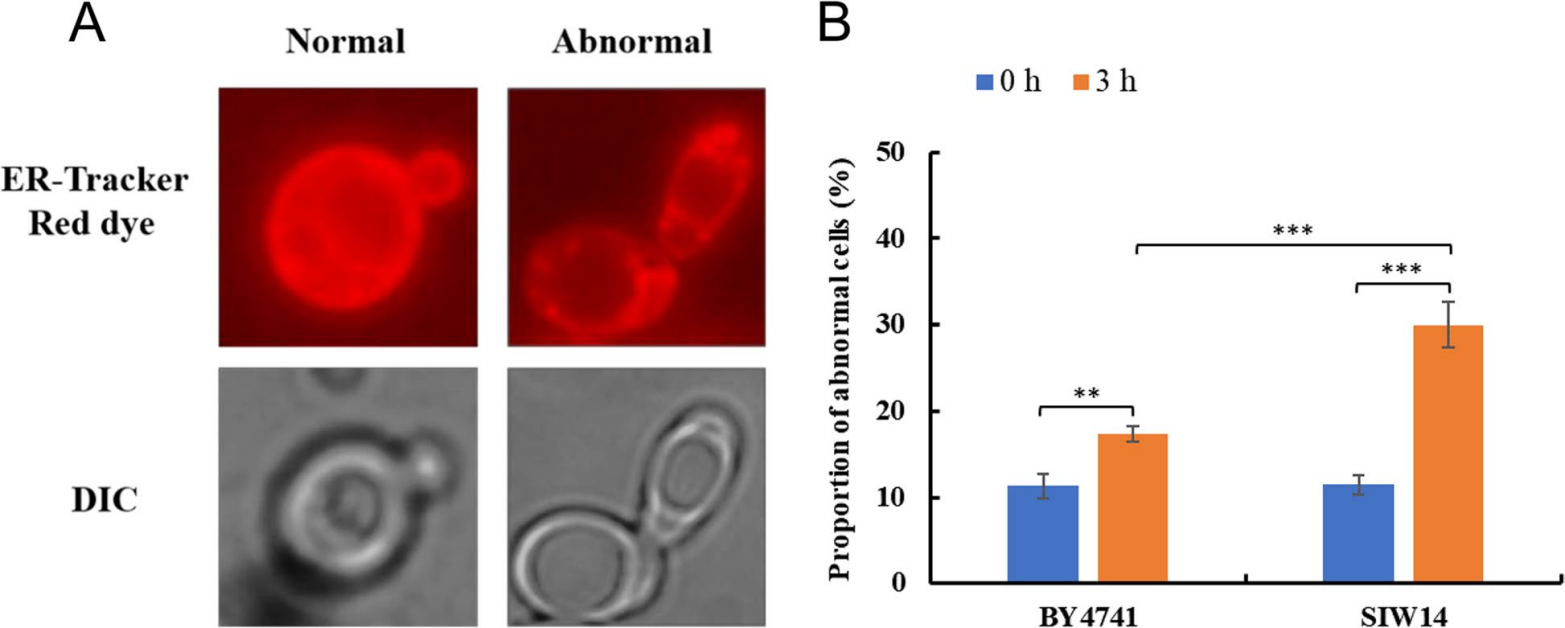
Morphological changes of endoplasmic reticulum in *S. cerevisiae.* (**A**) Different morphologies of the ER in cells. (**B**) The proportion of cells at 0 mM and 60 mM HMF that displayed abnormal endoplasmic reticulum after treatment for 0 and 3 h. ER-Tracker Red dye: Endoplasmic reticulum stain. DIC: differential interference microscope. ** *p* < 0.01, *** *p* < 0.001 indicates significant differences. The data represent averages of three experiments. At least 100 cells were examined on each bright-field image(Figure S2, S3)

Based on previous research [24], we classified the structural morphology of the endoplasmic reticulum in living cells into two categories, normal and abnormal (Fig. 7A). The percentage of cells with ER damage at 0 h was 11.3% for standard strain BY4741 (CK) and 11.12% for *Siw14Δ* (*SIW14* knockout strain), and the damage ratios were basically the same (Fig. 7B). However, after 3 h HMF treatment, the percentage of cells with ER damage increased in both strains, 17.32% in BY4741 and 30% in *Siw14Δ*, the latter significant higher than the former (Fig. 7B). It can be seen that HMF disrupts the structure of the endoplasmic reticulum, and has a more adverse effect on *Siw14Δ*.

### Vacuoles

**Fig. 8:**
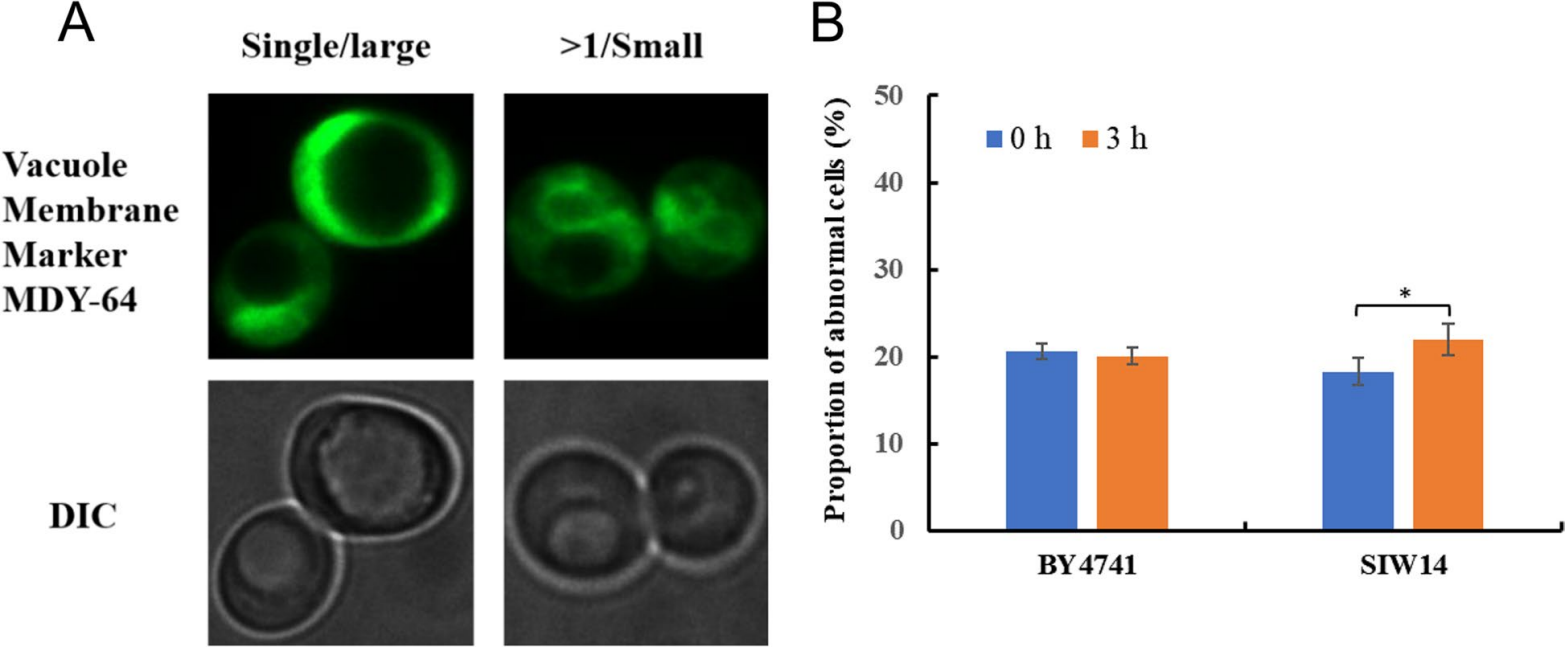
Morphological changes of vacuoles in *S. cerevisiae*. (**A**) Different morphology of vacuoles in cells. (**B**) The proportion of cells at 0 mM and 60 mM HMF that displayed abnormal vacuoles after treatment for 0 and 3 h. Vacuole Membrane Marker MDY-64: vacuole dyeing agent. DIC: differential interference microscope. Single/large: single large vacuole. > 1/ Small: more than single small vacuole. * *p* < 0.05 indicates significant differences. The data represent averages of three experiments. At least 100 cells were examined on each bright-field image(Figure S2, S3)

Two different vacuole morphologies were observed in this experiment, a single large vacuole and more than one fragmented vacuole (Fig. 8A). After 3 h HMF treatment, the structure of intracellular vacuoles saw no remarkable change in both strains. The percentage of cells with vacuole damage in standard strain BY4741 (CK) was 20.59% and 20.10% at 0 h and 3 h, and that in *Siw14Δ* (*SIW14* knockout strain) was 18.33% and 22%, respectively (Fig. 8B). In summary, the effect of HMF on the structure of intracellular vacuoles was not significant after the knockout of *SIW14* gene.

### Chromatin

**Fig. 9:**
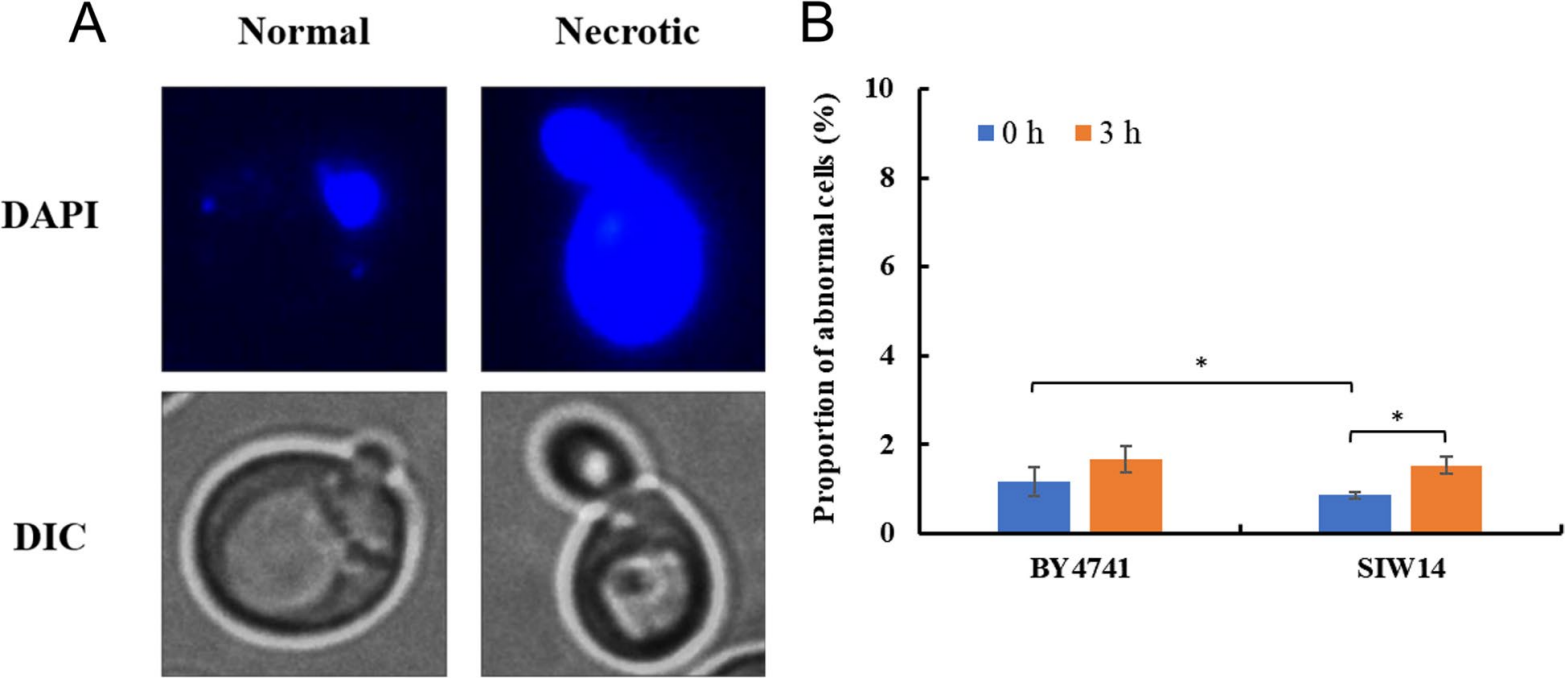
Morphological changes of chromatin in *S. cerevisiae*. (**A**) Different morphologies of chromatin in cells. (**B**) The proportion of cells at 0 mM and 60 mM HMF that displayed abnormal chromatin after treatment for 0 and 3 h. DAPI: DNA specific dye diamino phenylindole. DIC: differential interference microscope. * *p* < 0.05 indicates significant differences. The data represent averages of three experiments. At least 100 cells were examined on each bright-field image(Figure S2, S3)

The normal chromatin structure in the cell is small and dense, while cells with severe chromatin damage become necrotic and the entire cell is stained blue, indicating abnormal function of the cell (Fig. 9A). The percentage of BY4741 and *Siw14Δ* (*SIW14* knockout strain) containing abnormal cells at 0 h and 3 h—1.17% and 1.66% for the standard strain BY4741 (CK) was, and 0.86% and 1.54% for the Siw14Δ, respectively (Fig. 9B). It can be seen that the percentage of abnormal cells in both BY4741 and *Siw14Δ* remained low before and after HMF treatment, indicating that HMF will not destroy the structure of chromatin.

## Discussion

In our study, 202 sensitive genes and 92 tolerance genes were selected through the whole gene knockout library of HMF, and KEGG and GO enrichment analysis were carried out on the sensitive and tolerance genes using Cytoscape software, respectively. It was found that these genes were enriched in multiple pathways, such as membrane fluidity, protein phosphorylation, cytoplasmic sac and peroxysome tissue. Ma et al. identified *YAP1*, *PDR1*, *PDR3*, *RPN4* and *HSF1* as key regulatory genes in the tolerance of *S. cerevisiae* to lignocellulose-derived inhibitor HMF through transcriptomic analysis [26]. In this study, SGA scan revealed that deletion of *YAP1*, *PDR1* and *PDR3* genes did not change the tolerance of *S. cerevisiae* to HMF. *RPN4* gene deletion slightly reduced *S. cerevisiae*'s tolerance to HMF. Given that previous studies were conducted only based on transcriptome sequencing with results left unverified, the experimental results were significantly inaccurate. However, in this study, phenotype was used as a guide to analyze HMF tolerance genes by SGA scanning, which can fully explore the potential genes related to HMF tolerance in *S. cerevisiae*.

Since they are two furan inhibitors both containing aldehyde group and furan ring two major toxic groups, HMF and furfural show similar physiological toxicity. Therefore, the literature on whole gene knockout library with furfural treatment is also reviewed. According to the literature, 62 genes in *S. cerevisiae* were associated with furfural tolerance, and these genes were mainly concentrated in carbon metabolism, chromatin modification or mRNA transport, budding and cytokinesis, cytoskeletal function, DNA replication and damage repair pathways (e.g., *RPE1*, *EAR7*, *BUD27*, etc.) [16]. Compared with the HMF tolerance-related pathways described in this paper, it was found that carbon metabolism, chromatin modification or mRNA transport, germination and cytoplasmic division were common pathways related to furfural and HMF tolerance. However, the genes involved in the related pathways still differed greatly. In addition, our research found that some specific pathways unrelated to furfural tolerance played a role in HMF tolerance such as cytoplasmic sac, membrane permeability and protein phosphorylation pathways. This suggests that although the toxic mechanisms of HMF and furfural are similar, their tolerance mechanisms vary in *S. cerevisiae*.

Phosphorylation is a common post-translational modification of proteins that effect the biological functions such as protein interaction and intracellular localization [27]. In this study, two new HMF tolerance-related genes (*OCA1* and *SIW14*) were discovered, which are involved in the protein phosphorylation pathway, and the *SIW14* gene was confirmed to play a key role in HMF tolerance by dot plate tests. Wang et al. revealed the high activity and exquisite specificity of Siw14p, a member of the protein tyrosine phosphatase (PTP) superfamily, in the catalysis of the 5-diphosphate group of inositol pyrophosphate (PP-InsPs) [27]. PP-InsPs are diffused intracellular signaling molecules with unique and crowded arrays of multiple phosphates and "highly energetic" di-phosphonates, such as InsP6, 5-InsP7, InsP8, etc. Siw14p promotes intracellular con-version of 5-InsP7 to InsP6 and InsP8 to 1-InsP7 and ADP to ATP [27, 28]. Thus, the ab-sence of *SIW14* leads to a reduction in ATP synthesis during oxidative phosphorylation. When ATP is reduced, programmed cell death will be affected resulting in an increase and subsequent accumulation of intracellular ROS in the process [29]. This is consistent with the increase in intracellular ROS of *SIW14* knockout strain under HMF stress in our study.

The endoplasmic reticulum is an organelle that serves as the cell's manufacturing plant where protein misfolding or damage causes accumulation of misfolded or un-folded proteins, known as endoplasmic reticulum stress [24]. In an experiment to observe the structure of the ER, it was found that cells containing abnormal ER in *Siw14Δ* (*SIW14* knockout strain) increased by two times compared with that in BY4741 after 3 h HMF treatment. This may be triggered by the destruction of the membrane phospholipid bi-layer of the ER or by the accumulation of unfolded or misfolded proteins in the ER. The analysis of reactive oxygen species accumulation revealed that excessive accumulation of ROS was highly likely to impair lipids, proteins and other biomolecules on the ER reticulum, which can culminate in ER reticulum phospholipid layer damage and chronic ER stress [30, 31].

## Conclusion

Our study is a frontrunner in using the growth of the whole gene knockout library colony of *S. cerevisiae* as the phenotypic guide and clearly identified 202 sensitive genes and 92 tolerance genes related to 5-hydroxymethylfurfural tolerance with the help of bioinformatics analysis. The HMF tolerance genes *OCA1* and *SIW14* on the protein phosphorylation pathway were verified by the spot test and the subcellular structure observation test and *SIW14* gene is determined as a key gene for 5-hydroxymethylfurfural tolerance in *S. cerevisiae*. This discovery not only provides guidance for the later modification of *S. cerevisiae* with higher tolerance but also represents a significant contribution towards improving the fermentation efficiency of bioethanol production from lignocellulosic material. In order to further explore the tolerance mechanism of *S. cerevisiae* to 5-hydroxymethylfurfural, a toxic by-product of lignocellulosic hydrolysate, the validation study of remaining sensitivity or tolerance genes is required subsequently.

### Supplementary Information

**Additional file 1. Table S1.** SGAtool analyzed the scores of each knockout strain under HMF conditions.** Figure S1. **Growth curves of BY4741 and Siw14Δ in YPD+G418 liquid medium. **Figure S2.** The original image from the subcellular observation experiment with BY4741. (**A**) Reactive Oxygen species andMitochondria. (**B**) Endoplasmic reticulum and Vacuoles. (**C**) Chromatin.** Figure S3. **The original image from the subcellular observation experiment with Siw14Δ. (**A**) Reactive Oxygen species and Mitochondria. (**B**) Endoplasmic reticulum and Vacuoles. (**C**) Chromatin.

## Data Availability

The data used to support the fndings of this study are available from the corresponding author upon request.

## References

[1] Vohra M, Manwar J, Manmode R, Padgilwar S, Patil S (2014). Bioethanol production: feedstock and current technologies. J Environ Chem Eng.

[2] Zabed H, Faruq G, Sahu JN, Azirun MS, Hashim R, Amru NB. Bioethanol production from fermentable sugar juice. The Scientific World Joournal. 2014, 957. 10.1155/2014/957102 10.1155/2014/957102 PMC3970039 24715820

[3] Hu J, Lin Y, Zhang Z, Xiang T, Mei Y, Zhao S, Liang Y, Peng N (2016). High-titer lactic acid production by *Lactobacillus pentosus* FL0421 from corn stover using fed-batch simultaneous saccharification and fermentation. Biores Technol.

[4] Joe MH, Kim JY, Lim S, Kim DH, Bai S, Park H, Lee SG, Han SJ, Choi J (2015). Microalgal lipid production using the hydrolysates of rice straw pretreated with gamma irradiation and alkali solution. Biotechnol Biofuels.

[5] Reifenrath M, Boles E (2018). Engineering of hydroxymandelate synthases and the aromatic amino acid pathway enables de novo biosynthesis of mandelic and 4-hydroxymandelic acid with *Saccharomyces cerevisiae*. Metab Eng.

[6] Magnus A, Maurizio B, Valeria M, Lisbeth O (2013). The influence of HMF and furfural on redox-balance and energy-state of xylose-utilizing *Saccharomyces cerevisiae*. Biotechnol Biofuels.

[7] Taherzadeh MJ, Gustafsson L, Niklasson C, Lidén G (1999). Conversion of furfural in aerobic and anaerobic batch fermentation of glucose by *Saccharomyces cerevisiae*. J Biosci Bioeng.

[8] Allen SA, Clark W, McCaffery MJ, Cai Z, Lanctot A, Slininger PJ, Liu ZL, Gorsoch SW (2010). Furfural induces reactive oxygen species accumulation and cellular damage in *Saccharomyces cerevisiae*. Biotechnol Biofuels.

[9] Becerra ML, Lizarazo LM, Rojas HA, Prieto GA, Martinez JJ (2022). Biotransformation of 5-hydroxymethylfurfural and furfural with bacteria of *bacillus genus*. Biocatal Agric Biotechnol.

[10] Goffeau A, Barrell BG, Bussey H, Davis RW, Dujon B, Feldmann H, Galibert F, Hoheisel JD, Jacq C, Johnston M, Louis EJ (1996). Life with 6000 genes. Science.

[11] Giaever G, Chu AM, Ni L, Connelly C, Riles L, Véronneau S, Dow S, Lucau-Danila A, Anderson K, André B, Arkin AP (2002). Functional profiling of the *Saccharomyces cerevisiae* genome. Nature.

[12] Winzeler EA, Shoemaker DD, Astromoff A, Liang H, Anderson K, Andre B, Bangham R, Benito R, Boeke JD, Bussey H, Chu AM (1999). Functional characterization of the *Saccharomyces cerevisiae* genome by gene deletion and parallel analysis. Science.

[13] Nawaz-ul-Rehman MS, Prasanth KR, Baker J, Nagy PD (2013). Yeast screens for host factors in positive-strand RNA virus replication based on a library of temperature-sensitive mutants. Methods.

[14] Douglas AC, Smith AM, Sharifpoor S, Yan Z, Durbic T, Heisler LE, Lee AY, Ryan O, Göttert H, Surendra A, vanDyk, D. Functional analysis with a barcoder yeast gene overexpression system. G3 Genesgenetics. 2012;2:1279–1289. 10.1534/g3.112.003400 10.1534/g3.112.003400 PMC3464120 23050238

[15] Tkach JM, Yimit A, Lee AY, Riffle M, Costanzo M, Jaschob D, Hendry JA, Ou J, Moffat J, Boone C, Davis TN (2012). Dissecting DNA damage response pathways by analysing protein localization and abundance changes during DNA replication stress. Nat Cell Biol.

[16] Gorsich SW, Dien BS, Nichols NN, Slininger PJ, Liu ZL, Skory CD (2006). Tolerance to furfural-induced stress is associated with pentose phosphate pathway genes *ZWF1*, *GND1*, *RPE1*, and *TKL1* in *Saccharomyces cerevisiae*. Appl Microbiol Biotechnol.

[17] Tong AH, Evangelista M, Parsons AB, Xu H, Bader GD, Pagé N, Robinson M, Raghibizadeh S, Hogue CW, Bussey H, Andrews B (2001). Systematic genetic analysis with ordered arrays of yeast deletion mutants. Science..

[18] Hill SM, Hao X, Liu B, Nyström T (2014). Life-span extension by a metacaspase in the yeast *Saccharomyces cerevisiae*. Science.

[19] Hanzén S, Vielfort K, Yang J, Roger F, Andersson V, Zamarbide-Forés S, Andersson R, Malm L, Palais G, Biteau B, Liu B, Michel BT, Mikael M, Thomas N (2016). Lifespan control by redox-dependent recruitment of chaperones to misfolded proteins. Cell.

[20] Cao X, An T, Fu W, Zhang J, Zhao H, Li D, Jin X, Liu B (2022). Genome-wide identification of cellular pathways and key genes that respond to sodium bicarbonate stress in *Saccharomyces cerevisiae*. Front Microbiol.

[21] Wagih O, Usaj M, Baryshnikova A, VanderSluis B, Kuzmin E, Costanzo M, Myers CL, Andrews BJ, Boone CM, Parts L (2013). SGAtools: one-stop analysis and visualization of array-based genetic interaction screens. Nucleic Acids Res.

[22] Shannon P, Markiel A, Ozier O, Baliga NS, Wang JT, Ramage D, Amin N, Schwikowski B, Ideker T (2003). Cytoscape: a software environment for integrated models of biomolecular interaction networks. Genome Res..

[23] Madeo F, Fröhlich E, Ligr M, Grey M, Sigrist SJ, Wolf DH, Fröhlich KU (1999). Oxygen stress: A regulator of apoptosis in yeast. J Cell Biol..

[24] Wang HY, Li Q, Zhang ZY, Kuang XL, Hu XD, Ayepa E, Han XB, Abrha GT (2020). Cellular Analysis and Comparative Transcriptomics Reveal the Tolerance Mechanisms of *Candida tropicalis* Toward Phenol. Front Microbiol.

[25] Ellen SA, Clark W, McCaffery JM, Cai Z, Lanctot A, Slininger PJ, Liu ZL, Gorsich SW (2010). Furfural induces reactive oxygen species accumulation and cellular damage in *Saccharomyces cerevisiae*. Biotechnol Biofuels.

[26] Ma MG, Liu ZL (2010). Comparative transcriptome profiling analyses during the lag phase uncover *YAP1*, *PDR1*, *PDR3*, *RPN4*, and *HSF1* as key regulatory genes in genomic adaptation to the lignocellulose derived inhibitor HMF for *Saccharomyces cerevisiae*. BMC Genomics.

[27] Wang HC, Gu CF, Rolfes RJ, Jessen HJ, Shears SB (2018). Structural and biochemical characterization of Siw14: A protein-tyrosine phosphatase fold that metabolizes inositol pyrophosphates. J Biol Chem.

[28] Gu CF, Nguyen HN, Douglas G, Chen ZW, Jessen HJ, Gu Z, Wang HC, Shears SB (2017). KO of 5-InsP_7_ kinase activity transforms the HCT116 colon cancer cell line into a hypermetabolic, growth-inhibited phenotype. Proc Natl Acad Sci USA.

[29] Brookes PS, Yoon Y, Robotham JL, Anders MW, Sheu SS (2004). Calcium, ATP, and ROS: a mitochondrial love-hate triangle. Am J Physiol Cell Physiol..

[30] Perrone GG, Tan SX, Dawes IW (2008). Reactive oxygen species and yeast apoptosis. Biochim Biophys Acta.

[31] Senft D, Ronai Z (2015). UPR, autophagy, and mitochondria crosstalk underlies the ER stress response. Trends Biochem Sci.

