# Supplementary material for: Genome-wide identification of resistance genes and cellular analysis of key gene knockout strain under 5-hydroxymethylfurfural stress in Saccharomyces cerevisiae
Source: BMC Microbiol. 2023 Dec 4;23:382. doi: 10.1186/s12866-023-03095-2 (PMC10694885; doi:10.1186/s12866-023-03095-2)
Supplement: Supplementary file 1 — Additional file 1. Table S1. SGAtool analyzed the scores of each knockout strain under HMF conditions. Figure S1. Growth curves of BY4741 and Siw14Δ in YPD+G418 liquid medium. Figure S2. The original image from the subcellular observation experiment with BY4741. (A) Reactive Oxygen species andMitochondria. (B) Endoplasmic reticulum and Vacuoles. (C) Chromatin. Figure S3. The original image from the subcellular observation experiment with Siw14Δ. (A) Reactive Oxygen species and Mitochondria. (B) Endoplasmic reticulum and Vacuoles. (C) Chromatin. [file 12866_2023_3095_MOESM1_ESM.docx]

**Table S1. SGAtool analyzed the scores of each knockout strain under HMF conditions**

| NO | Array ORF | Array Name | Normalized colony size (EXPERIMENT) | Normalized colony std. dev. (EXPERIMENT) | Normalized colony size (CONTROL) | Normalized colony std. dev. (CONTROL) | Score | Score stdev | p-Value |
| --- | --- | --- | --- | --- | --- | --- | --- | --- | --- |
| 1 | *YMR283C* | *RIT1* | 0.38 | 0.01 | 1.04 | 0.03 | -0.66 | 0.01 | 0.00 |
| 2 | *YDR049W* | *VMS1* | 0.38 | 0.00 | 0.98 | 0.02 | -0.60 | 0.00 | 0.00 |
| 3 | *YJR075W* | *HOC1* | 0.69 | 0.01 | 1.21 | 0.02 | -0.52 | 0.01 | 0.00 |
| 4 | *YBL047C* | *EDE1* | 0.40 | 0.02 | 0.92 | 0.01 | -0.52 | 0.02 | 0.00 |
| 5 | *YDR068W* | *DOS2* | 0.42 | 0.04 | 0.94 | 0.02 | -0.52 | 0.04 | 0.00 |
| 6 | *YGL105W* | *ARC1* | 0.27 | 0.01 | 0.78 | 0.00 | -0.51 | 0.01 | 0.00 |
| 7 | *YHR030C* | *SLT2* | 0.80 | 0.05 | 1.29 | 0.05 | -0.48 | 0.05 | 0.00 |
| 8 | *YOR191W* | *ULS1* | 0.65 | 0.02 | 1.11 | 0.03 | -0.46 | 0.02 | 0.00 |
| 9 | *YMR272C* | *SCS7* | 0.61 | 0.07 | 1.06 | 0.02 | -0.45 | 0.07 | 0.00 |
| 10 | *YJL124C* | *LSM1* | 0.43 | 0.02 | 0.88 | 0.03 | -0.45 | 0.02 | 0.00 |
| 11 | *YDR360W* | *YDR360W* | 0.62 | 0.02 | 1.05 | 0.00 | -0.43 | 0.02 | 0.00 |
| 12 | *YNL032W* | *SIW14* | 0.72 | 0.02 | 1.12 | 0.12 | -0.42 | 0.02 | 0.00 |
| 13 | *YML035C* | *AMD1* | 0.51 | 0.01 | 0.93 | 0.04 | -0.41 | 0.01 | 0.00 |
| 14 | *YBR025C* | *OLA1* | 0.55 | 0.01 | 0.96 | 0.01 | -0.41 | 0.01 | 0.00 |
| 15 | *YDR378C* | *LSM6* | 0.29 | 0.02 | 0.69 | 0.03 | -0.40 | 0.02 | 0.00 |
| 16 | *YNR051C* | *BRE5* | 0.64 | 0.00 | 1.05 | 0.02 | -0.40 | 0.00 | 0.00 |
| 17 | *YLR217W* | *YLR217W* | 0.94 | 0.02 | 1.35 | 0.03 | -0.39 | 0.02 | 0.00 |
| 18 | *YJL121C* | *RPE1* | 0.37 | 0.05 | 0.75 | 0.05 | -0.39 | 0.05 | 0.00 |
| 19 | *YMR275C* | *BUL1* | 0.66 | 0.03 | 1.02 | 0.04 | -0.37 | 0.03 | 0.00 |
| 20 | *YKL023W* | *YKL023W* | 0.64 | 0.04 | 1.01 | 0.03 | -0.36 | 0.04 | 0.00 |
| 21 | *YNR005C* | *YNR005C* | 0.74 | 0.01 | 1.09 | 0.06 | -0.35 | 0.01 | 0.00 |
| 22 | *YLR085C* | *ARP6* | 0.67 | 0.00 | 1.03 | 0.04 | -0.34 | 0.00 | 0.00 |
| 23 | *YML041C* | *VPS71* | 0.63 | 0.04 | 0.98 | 0.04 | -0.34 | 0.04 | 0.00 |
| 24 | *YJR059W* | *PTK2* | 0.49 | 0.04 | 0.83 | 0.02 | -0.34 | 0.04 | 0.00 |
| 25 | *YNL136W* | *EAF7* | 0.45 | 0.02 | 0.78 | 0.03 | -0.33 | 0.02 | 0.00 |
| 26 | *YJL136C* | *RPS21B* | 0.60 | 0.06 | 0.92 | 0.02 | -0.32 | 0.06 | 0.00 |
| 27 | *YLR330W* | *CHS5* | 0.85 | 0.03 | 1.16 | 0.04 | -0.32 | 0.03 | 0.00 |
| 28 | *YCR053W* | *THR4* | 0.65 | 0.02 | 0.97 | 0.01 | -0.31 | 0.02 | 0.00 |
| 29 | *YNL099C* | *OCA1* | 0.78 | 0.01 | 1.09 | 0.03 | -0.31 | 0.01 | 0.00 |
| 30 | *YGL084C* | *GUP1* | 0.51 | 0.01 | 0.80 | 0.01 | -0.30 | 0.01 | 0.00 |
| 31 | *YDL078C* | *MDH3* | 0.91 | 0.03 | 1.21 | 0.04 | -0.30 | 0.03 | 0.00 |
| 32 | *YOR006C* | *TSR3* | 0.86 | 0.05 | 1.16 | 0.06 | -0.30 | 0.05 | 0.00 |
| 33 | *YDR067C* | *OCA6* | 0.73 | 0.02 | 1.03 | 0.03 | -0.30 | 0.02 | 0.00 |
| 34 | *YER151C* | *UBP3* | 0.73 | 0.01 | 1.02 | 0.01 | -0.29 | 0.01 | 0.00 |
| 35 | *YNL229C* | *URE2* | 0.69 | 0.02 | 0.98 | 0.01 | -0.29 | 0.02 | 0.00 |

**Table S1. SGAtool analyzed the scores of each knockout strain under HMF conditions (Continue)**

| NO | Array ORF | Array Name | Normalized colony size (EXPERIMENT) | Normalized colony std. dev. (EXPERIMENT) | Normalized colony size (CONTROL) | Normalized colony std. dev. (CONTROL) | Score | Score stdev | p-Value |
| --- | --- | --- | --- | --- | --- | --- | --- | --- | --- |
| 36 | *YPR134W* | *MSS18* | 0.55 | 0.02 | 0.84 | 0.00 | -0.29 | 0.02 | 0.00 |
| 37 | *YPR024W* | *YME1* | 0.56 | 0.01 | 0.84 | 0.02 | -0.28 | 0.01 | 0.00 |
| 38 | *YKL046C* | *DCW1* | 0.94 | 0.09 | 1.22 | 0.05 | -0.28 | 0.09 | 0.00 |
| 39 | *YHR086W* | *NAM8* | 0.75 | 0.04 | 1.02 | 0.03 | -0.28 | 0.04 | 0.00 |
| 40 | *YDL050C* | *YDL050C* | 0.74 | 0.00 | 1.01 | 0.01 | -0.27 | 0.00 | 0.00 |
| 41 | *YML008C* | *ERG6* | 0.50 | 0.02 | 0.77 | 0.02 | -0.27 | 0.02 | 0.00 |
| 42 | *YNL147W* | *LSM7* | 0.51 | 0.05 | 0.78 | 0.04 | -0.27 | 0.05 | 0.00 |
| 43 | *YGR038W* | *ORM1* | 0.93 | 0.01 | 1.20 | 0.03 | -0.26 | 0.01 | 0.00 |
| 44 | *YNL254C* | *RTC4* | 0.82 | 0.05 | 1.08 | 0.02 | -0.26 | 0.05 | 0.00 |
| 45 | *YPL183C* | *RTT10* | 0.73 | 0.00 | 0.99 | 0.02 | -0.26 | 0.00 | 0.00 |
| 46 | *YMR026C* | *PEX12* | 0.78 | 0.03 | 1.02 | 0.05 | -0.25 | 0.03 | 0.00 |
| 47 | *YBL094C* | *YBL094C* | 0.71 | 0.01 | 0.96 | 0.07 | -0.25 | 0.01 | 0.00 |
| 48 | *YDR485C* | *VPS72* | 0.67 | 0.01 | 0.92 | 0.00 | -0.25 | 0.01 | 0.00 |
| 49 | *YJL062W* | *LAS21* | 0.74 | 0.02 | 0.99 | 0.01 | -0.25 | 0.02 | 0.00 |
| 50 | *YOR012W* | *YOR012W* | 0.76 | 0.02 | 1.00 | 0.01 | -0.25 | 0.02 | 0.00 |
| 51 | *YHR078W* | *YHR078W* | 0.85 | 0.03 | 1.09 | 0.01 | -0.24 | 0.03 | 0.00 |
| 52 | *YNL008C* | *ASI3* | 0.95 | 0.03 | 1.19 | 0.05 | -0.24 | 0.03 | 0.00 |
| 53 | *YFL023W* | *BUD27* | 0.64 | 0.03 | 0.88 | 0.03 | -0.24 | 0.03 | 0.00 |
| 54 | *YJR082C* | *EAF6* | 0.78 | 0.02 | 1.02 | 0.02 | -0.24 | 0.02 | 0.00 |
| 55 | *YNL072W* | *RNH201* | 0.76 | 0.03 | 1.00 | 0.02 | -0.24 | 0.03 | 0.00 |
| 56 | *YMR282C* | *AEP2* | 0.78 | 0.02 | 1.01 | 0.04 | -0.23 | 0.02 | 0.00 |
| 57 | *YLR452C* | *SST2* | 0.99 | 0.02 | 1.23 | 0.02 | -0.23 | 0.02 | 0.00 |
| 58 | *YBR001C* | *NTH2* | 0.74 | 0.02 | 0.97 | 0.00 | -0.23 | 0.02 | 0.00 |
| 59 | *YNL089C* | *YNL089C* | 0.85 | 0.02 | 1.08 | 0.02 | -0.23 | 0.02 | 0.00 |
| 60 | *YMR031W-A* | *YMR031W-A* | 0.59 | 0.03 | 0.83 | 0.03 | -0.23 | 0.03 | 0.00 |
| 61 | *YLR332W* | *MID2* | 0.92 | 0.05 | 1.14 | 0.04 | -0.23 | 0.05 | 0.00 |
| 62 | *YHR066W* | *SSF1* | 0.77 | 0.01 | 1.00 | 0.01 | -0.23 | 0.01 | 0.00 |
| 63 | *YBL007C* | *SLA1* | 0.64 | 0.01 | 0.87 | 0.03 | -0.23 | 0.01 | 0.00 |
| 64 | *YJR074W* | *MOG1* | 0.64 | 0.05 | 0.87 | 0.03 | -0.23 | 0.05 | 0.00 |
| 65 | *YDR304C* | *CPR5* | 0.88 | 0.01 | 1.11 | 0.03 | -0.23 | 0.01 | 0.00 |
| 66 | *YLR098C* | *CHA4* | 0.89 | 0.01 | 1.12 | 0.04 | -0.23 | 0.01 | 0.00 |
| 67 | *YIL132C* | *CSM2* | 0.91 | 0.07 | 1.13 | 0.03 | -0.22 | 0.07 | 0.00 |
| 68 | *YLR070C* | *XYL2* | 0.94 | 0.06 | 1.17 | 0.05 | -0.22 | 0.06 | 0.00 |
| 69 | *YMR318C* | *ADH6* | 0.81 | 0.02 | 1.03 | 0.02 | -0.22 | 0.02 | 0.00 |
| 70 | *YDR532C* | *KRE28* | 0.57 | 0.01 | 0.79 | 0.02 | -0.22 | 0.01 | 0.00 |

**Table S1. SGAtool analyzed the scores of each knockout strain under HMF conditions (Continue)**

| NO | Array ORF | Array Name | Normalized colony size (EXPERIMENT) | Normalized colony std. dev. (EXPERIMENT) | Normalized colony size (CONTROL) | Normalized colony std. dev. (CONTROL) | Score | Score stdev | p-Value |
| --- | --- | --- | --- | --- | --- | --- | --- | --- | --- |
| 71 | *YJR097W* | *JJJ3* | 0.70 | 0.03 | 0.93 | 0.03 | -0.22 | 0.03 | 0.00 |
| 72 | *YJL211C* | *YJL211C* | 0.89 | 0.04 | 1.10 | 0.05 | -0.22 | 0.04 | 0.00 |
| 73 | *YGR051C* | *YGR051C* | 0.97 | 0.02 | 1.19 | 0.11 | -0.22 | 0.02 | 0.00 |
| 74 | *YFL013C* | *IES1* | 0.87 | 0.03 | 1.09 | 0.05 | -0.22 | 0.03 | 0.00 |
| 75 | *YDR334W* | *SWR1* | 0.72 | 0.01 | 0.94 | 0.03 | -0.22 | 0.01 | 0.00 |
| 76 | *YHR110W* | *ERP5* | 0.90 | 0.02 | 1.12 | 0.00 | -0.22 | 0.02 | 0.00 |
| 77 | *YHL024W* | *RIM4* | 0.92 | 0.01 | 1.13 | 0.01 | -0.21 | 0.01 | 0.00 |
| 78 | *YGR055W* | *MUP1* | 0.62 | 0.02 | 0.84 | 0.04 | -0.21 | 0.02 | 0.00 |
| 79 | *YCL009C* | *ILV6* | 0.94 | 0.04 | 1.15 | 0.04 | -0.21 | 0.04 | 0.00 |
| 80 | *YLL046C* | *RNP1* | 0.98 | 0.00 | 1.21 | 0.10 | -0.21 | 0.00 | 0.00 |
| 81 | *YCR085W* | *YCR085W* | 0.81 | 0.02 | 1.01 | 0.02 | -0.21 | 0.02 | 0.00 |
| 82 | *YHL023C* | *NPR3* | 0.83 | 0.01 | 1.04 | 0.03 | -0.21 | 0.01 | 0.00 |
| 83 | *YJL141C* | *YAK1* | 0.84 | 0.10 | 1.04 | 0.03 | -0.21 | 0.10 | 0.00 |
| 84 | *YCR062W* | *YCR062W* | 0.86 | 0.03 | 1.08 | 0.03 | -0.21 | 0.03 | 0.00 |
| 85 | *YPL213W* | *LEA1* | 0.67 | 0.01 | 0.88 | 0.02 | -0.20 | 0.01 | 0.00 |
| 86 | *YGL059W* | *PKP2* | 0.97 | 0.04 | 1.18 | 0.01 | -0.20 | 0.04 | 0.00 |
| 87 | *YBL024W* | *NCL1* | 0.77 | 0.02 | 0.97 | 0.00 | -0.20 | 0.02 | 0.00 |
| 88 | *YKR084C* | *HBS1* | 0.67 | 0.03 | 0.88 | 0.02 | -0.20 | 0.03 | 0.00 |
| 89 | *YPL105C* | *SYH1* | 0.89 | 0.01 | 1.09 | 0.01 | -0.20 | 0.01 | 0.00 |
| 90 | *YGR122C-A* | *YGR122C-A* | 0.98 | 0.02 | 1.19 | 0.04 | -0.20 | 0.02 | 0.00 |
| 91 | *YDL051W* | *LHP1* | 0.80 | 0.02 | 1.00 | 0.01 | -0.20 | 0.02 | 0.00 |
| 92 | *YMR163C* | *INP2* | 0.81 | 0.01 | 1.01 | 0.02 | -0.20 | 0.01 | 0.00 |
| 93 | *YDL091C* | *UBX3* | 1.21 | 0.03 | 1.01 | 0.01 | 0.20 | 0.03 | 0.00 |
| 94 | *YKR019C* | *IRS4* | 1.19 | 0.05 | 0.99 | 0.01 | 0.20 | 0.05 | 0.00 |
| 95 | *YGL014W* | *PUF4* | 1.24 | 0.05 | 1.04 | 0.01 | 0.20 | 0.05 | 0.00 |
| 96 | *YOR108W* | *LEU9* | 1.15 | 0.05 | 0.94 | 0.01 | 0.20 | 0.05 | 0.00 |
| 97 | *YDR032C* | *PST2* | 1.17 | 0.03 | 0.97 | 0.02 | 0.20 | 0.03 | 0.00 |
| 98 | *YJR014W* | *TMA22* | 1.11 | 0.01 | 0.90 | 0.02 | 0.20 | 0.01 | 0.00 |
| 99 | *YOL081W* | *IRA2* | 1.03 | 0.08 | 0.82 | 0.02 | 0.20 | 0.08 | 0.00 |
| 100 | *YIL085C* | *KTR7* | 1.03 | 0.01 | 0.83 | 0.01 | 0.20 | 0.01 | 0.00 |
| 101 | *YER066W* | *RRT13* | 1.17 | 0.05 | 0.97 | 0.01 | 0.20 | 0.05 | 0.00 |
| 102 | *YGR183C* | *QCR9* | 1.07 | 0.01 | 0.86 | 0.04 | 0.20 | 0.01 | 0.00 |
| 103 | *YGL180W* | *ATG1* | 1.22 | 0.06 | 1.01 | 0.03 | 0.21 | 0.06 | 0.00 |
| 104 | *YLR177W* | *YLR177W* | 1.14 | 0.01 | 0.93 | 0.02 | 0.21 | 0.01 | 0.00 |
| 105 | *YCR009C* | *RVS161* | 1.05 | 0.03 | 0.84 | 0.01 | 0.21 | 0.03 | 0.00 |

**Table S1. SGAtool analyzed the scores of each knockout strain under HMF conditions (Continue)**

| NO | Array ORF | Array Name | Normalized colony size (EXPERIMENT) | Normalized colony std. dev. (EXPERIMENT) | Normalized colony size (CONTROL) | Normalized colony std. dev. (CONTROL) | Score | Score stdev | p-Value |
| --- | --- | --- | --- | --- | --- | --- | --- | --- | --- |
| 106 | *YKR031C* | *SPO14* | 1.23 | 0.02 | 1.02 | 0.00 | 0.21 | 0.02 | 0.00 |
| 107 | *YML026C* | *RPS18B* | 1.04 | 0.02 | 0.84 | 0.02 | 0.21 | 0.02 | 0.00 |
| 108 | *YLR407W* | *YLR407W* | 1.08 | 0.00 | 0.87 | 0.03 | 0.21 | 0.00 | 0.00 |
| 109 | *YJR010C-A* | *SPC1* | 1.24 | 0.06 | 1.03 | 0.01 | 0.22 | 0.06 | 0.00 |
| 110 | *YBL104C* | *SEA4* | 1.11 | 0.01 | 0.89 | 0.01 | 0.22 | 0.01 | 0.00 |
| 111 | *YDL232W* | *OST4* | 0.91 | 0.05 | 0.70 | 0.01 | 0.22 | 0.05 | 0.00 |
| 112 | *YIL138C* | *TPM2* | 1.27 | 0.02 | 1.06 | 0.03 | 0.22 | 0.02 | 0.00 |
| 113 | *YGR105W* | *VMA21* | 1.04 | 0.02 | 0.83 | 0.02 | 0.22 | 0.02 | 0.00 |
| 114 | *YGR161C* | *RTS3* | 1.12 | 0.01 | 0.90 | 0.01 | 0.22 | 0.01 | 0.00 |
| 115 | *YBR141C* | *YBR141C* | 1.19 | 0.01 | 0.97 | 0.01 | 0.22 | 0.01 | 0.00 |
| 116 | *YKL146W* | *AVT3* | 1.15 | 0.02 | 0.94 | 0.02 | 0.22 | 0.02 | 0.00 |
| 117 | *YLR179C* | *YLR179C* | 1.26 | 0.04 | 1.05 | 0.04 | 0.22 | 0.04 | 0.00 |
| 118 | *YBR082C* | *UBC4* | 1.14 | 0.02 | 0.92 | 0.01 | 0.22 | 0.02 | 0.00 |
| 119 | *YEL057C* | *YEL057C* | 1.16 | 0.02 | 0.94 | 0.01 | 0.22 | 0.02 | 0.00 |
| 120 | *YCL036W* | *GFD2* | 1.20 | 0.04 | 0.98 | 0.01 | 0.22 | 0.04 | 0.00 |
| 121 | *YDR209C* | *YDR209C* | 1.30 | 0.01 | 1.08 | 0.03 | 0.22 | 0.01 | 0.00 |
| 122 | *YKL073W* | *LHS1* | 0.97 | 0.23 | 0.72 | 0.15 | 0.22 | 0.23 | 0.00 |
| 123 | *YKL048C* | *ELM1* | 0.92 | 0.06 | 0.70 | 0.02 | 0.22 | 0.06 | 0.00 |
| 124 | *YML004C* | *GLO1* | 1.21 | 0.01 | 0.98 | 0.01 | 0.22 | 0.01 | 0.00 |
| 125 | *YLR371W* | *ROM2* | 1.15 | 0.04 | 0.93 | 0.01 | 0.22 | 0.04 | 0.00 |
| 126 | *YOR183W* | *FYV12* | 1.10 | 0.01 | 0.87 | 0.01 | 0.23 | 0.01 | 0.00 |
| 127 | *YLR176C* | *RFX1* | 1.27 | 0.03 | 1.05 | 0.01 | 0.23 | 0.03 | 0.00 |
| 128 | *YAL022C* | *FUN26* | 1.17 | 0.09 | 0.92 | 0.07 | 0.23 | 0.09 | 0.00 |
| 129 | *YKR020W* | *VPS51* | 1.28 | 0.03 | 1.05 | 0.01 | 0.23 | 0.03 | 0.00 |
| 130 | *YHR162W* | *MPC2* | 1.02 | 0.04 | 0.79 | 0.02 | 0.23 | 0.04 | 0.00 |
| 131 | *YKL174C* | *TPO5* | 1.21 | 0.03 | 0.98 | 0.02 | 0.23 | 0.03 | 0.00 |
| 132 | *YLR384C* | *IKI3* | 1.10 | 0.02 | 0.87 | 0.00 | 0.23 | 0.02 | 0.00 |
| 133 | *YOR193W* | *PEX27* | 1.18 | 0.01 | 0.95 | 0.01 | 0.23 | 0.01 | 0.00 |
| 134 | *YNL171C* | *YNL171C* | 1.05 | 0.01 | 0.82 | 0.02 | 0.23 | 0.01 | 0.00 |
| 135 | *YDR508C* | *GNP1* | 1.26 | 0.02 | 1.03 | 0.01 | 0.23 | 0.02 | 0.00 |
| 136 | *YPR018W* | *RLF2* | 1.12 | 0.04 | 0.89 | 0.02 | 0.23 | 0.04 | 0.00 |
| 137 | *YIR019C* | *FLO11* | 1.25 | 0.10 | 1.03 | 0.04 | 0.23 | 0.10 | 0.00 |
| 138 | *YLR443W* | *ECM7* | 1.14 | 0.00 | 0.92 | 0.03 | 0.23 | 0.00 | 0.00 |
| 139 | *YNL170W* | *YNL170W* | 0.90 | 0.03 | 0.66 | 0.02 | 0.23 | 0.03 | 0.00 |
| 140 | *YML097C* | *VPS9* | 1.17 | 0.01 | 0.93 | 0.02 | 0.23 | 0.01 | 0.00 |

**Table S1. SGAtool analyzed the scores of each knockout strain under HMF conditions (Continue)**

| NO | Array ORF | Array Name | Normalized colony size (EXPERIMENT) | Normalized colony std. dev. (EXPERIMENT) | Normalized colony size (CONTROL) | Normalized colony std. dev. (CONTROL) | Score | Score stdev | p-Value |
| --- | --- | --- | --- | --- | --- | --- | --- | --- | --- |
| 141 | *YGR078C* | *PAC10* | 1.10 | 0.03 | 0.88 | 0.05 | 0.23 | 0.03 | 0.00 |
| 142 | *YOR182C* | *RPS30B* | 1.12 | 0.01 | 0.88 | 0.01 | 0.23 | 0.01 | 0.00 |
| 143 | *YMR145C* | *NDE1* | 1.23 | 0.07 | 0.99 | 0.02 | 0.23 | 0.07 | 0.00 |
| 144 | *YDL076C* | *RXT3* | 1.19 | 0.00 | 0.96 | 0.04 | 0.24 | 0.00 | 0.00 |
| 145 | *YKR047W* | *YKR047W* | 1.22 | 0.02 | 0.97 | 0.04 | 0.24 | 0.02 | 0.00 |
| 146 | *YMR029C* | *FAR8* | 1.19 | 0.04 | 0.96 | 0.02 | 0.24 | 0.04 | 0.00 |
| 147 | *YBR095C* | *RXT2* | 1.11 | 0.02 | 0.87 | 0.00 | 0.24 | 0.02 | 0.00 |
| 148 | *YNR029C* | *YNR029C* | 1.10 | 0.01 | 0.86 | 0.04 | 0.24 | 0.01 | 0.00 |
| 149 | *YMR271C* | *URA10* | 1.21 | 0.07 | 0.96 | 0.04 | 0.24 | 0.07 | 0.00 |
| 150 | *YLR327C* | *TMA10* | 1.16 | 0.03 | 0.91 | 0.03 | 0.24 | 0.03 | 0.00 |
| 151 | *YGL149W* | *YGL149W* | 1.21 | 0.02 | 0.98 | 0.02 | 0.24 | 0.02 | 0.00 |
| 152 | *YKL164C* | *PIR1* | 1.16 | 0.06 | 0.93 | 0.05 | 0.24 | 0.06 | 0.00 |
| 153 | *YML128C* | *MSC1* | 1.24 | 0.02 | 1.00 | 0.02 | 0.24 | 0.02 | 0.00 |
| 154 | *YDR156W* | *RPA14* | 1.34 | 0.03 | 1.10 | 0.03 | 0.24 | 0.03 | 0.00 |
| 155 | *YDL074C* | *BRE1* | 0.97 | 0.00 | 0.73 | 0.03 | 0.24 | 0.00 | 0.00 |
| 156 | *YMR039C* | *SUB1* | 1.28 | 0.08 | 1.04 | 0.02 | 0.24 | 0.08 | 0.00 |
| 157 | *YBR105C* | *VID24* | 0.95 | 0.01 | 0.71 | 0.00 | 0.24 | 0.01 | 0.00 |
| 158 | *YPL086C* | *ELP3* | 1.04 | 0.04 | 0.80 | 0.00 | 0.25 | 0.04 | 0.00 |
| 159 | *YDR469W* | *SDC1* | 1.09 | 0.01 | 0.85 | 0.01 | 0.25 | 0.01 | 0.00 |
| 160 | *YKL092C* | *BUD2* | 1.20 | 0.03 | 0.95 | 0.01 | 0.25 | 0.03 | 0.00 |
| 161 | *YPL071C* | *YPL071C* | 1.04 | 0.02 | 0.80 | 0.01 | 0.25 | 0.02 | 0.00 |
| 162 | *YLR287C-A* | *RPS30A* | 1.17 | 0.04 | 0.92 | 0.03 | 0.25 | 0.04 | 0.00 |
| 163 | *YOR078W* | *BUD21* | 1.04 | 0.01 | 0.79 | 0.01 | 0.25 | 0.01 | 0.00 |
| 164 | *YBR181C* | *RPS6B* | 1.18 | 0.00 | 0.93 | 0.01 | 0.25 | 0.00 | 0.00 |
| 165 | *YKR023W* | *YKR023W* | 1.21 | 0.01 | 0.97 | 0.01 | 0.25 | 0.01 | 0.00 |
| 166 | *YLR015W* | *BRE2* | 1.16 | 0.03 | 0.91 | 0.00 | 0.25 | 0.03 | 0.00 |
| 167 | *YMR312W* | *ELP6* | 1.07 | 0.02 | 0.82 | 0.02 | 0.25 | 0.02 | 0.00 |
| 168 | *YMR152W* | *YIM1* | 1.16 | 0.05 | 0.91 | 0.01 | 0.25 | 0.05 | 0.00 |
| 169 | *YGL179C* | *TOS3* | 1.22 | 0.02 | 0.97 | 0.01 | 0.25 | 0.02 | 0.00 |
| 170 | *YFL030W* | *AGX1* | 1.12 | 0.03 | 0.86 | 0.03 | 0.26 | 0.03 | 0.00 |
| 171 | *YKL109W* | *HAP4* | 1.11 | 0.05 | 0.86 | 0.01 | 0.26 | 0.05 | 0.00 |
| 172 | *YHR200W* | *RPN10* | 1.16 | 0.06 | 0.90 | 0.01 | 0.26 | 0.06 | 0.00 |
| 173 | *YIL154C* | *IMP2'* | 1.23 | 0.01 | 0.97 | 0.01 | 0.26 | 0.01 | 0.00 |
| 174 | *YLR024C* | *UBR2* | 1.21 | 0.04 | 0.95 | 0.03 | 0.26 | 0.04 | 0.00 |
| 175 | *YNL224C* | *SQS1* | 1.19 | 0.02 | 0.93 | 0.03 | 0.26 | 0.02 | 0.00 |

**Table S1. SGAtool analyzed the scores of each knockout strain under HMF conditions (Continue)**

| NO | Array ORF | Array Name | Normalized colony size (EXPERIMENT) | Normalized colony std. dev. (EXPERIMENT) | Normalized colony size (CONTROL) | Normalized colony std. dev. (CONTROL) | Score | Score stdev | p-Value |
| --- | --- | --- | --- | --- | --- | --- | --- | --- | --- |
| 176 | *YML035C-A* | *YML035C-A* | 1.24 | 0.02 | 0.98 | 0.02 | 0.26 | 0.02 | 0.00 |
| 177 | *YHR194W* | *MDM31* | 0.93 | 0.01 | 0.67 | 0.02 | 0.26 | 0.01 | 0.00 |
| 178 | *YDR459C* | *PFA5* | 1.14 | 0.02 | 0.88 | 0.04 | 0.26 | 0.02 | 0.00 |
| 179 | *YMR067C* | *UBX4* | 1.08 | 0.04 | 0.82 | 0.02 | 0.26 | 0.04 | 0.00 |
| 180 | *YLR320W* | *MMS22* | 1.07 | 0.01 | 0.81 | 0.01 | 0.26 | 0.01 | 0.00 |
| 181 | *YDR466W* | *PKH3* | 1.17 | 0.01 | 0.91 | 0.02 | 0.26 | 0.01 | 0.00 |
| 182 | *YHR059W* | *FYV4* | 1.02 | 0.05 | 0.76 | 0.01 | 0.26 | 0.05 | 0.00 |
| 183 | *YDL189W* | *RBS1* | 1.26 | 0.01 | 1.00 | 0.03 | 0.26 | 0.01 | 0.00 |
| 184 | *YDR096W* | *GIS1* | 1.33 | 0.01 | 1.07 | 0.03 | 0.26 | 0.01 | 0.00 |
| 185 | *YIL141W* | *YIL141W* | 1.26 | 0.01 | 0.99 | 0.03 | 0.26 | 0.01 | 0.00 |
| 186 | *YPL196W* | *OXR1* | 1.25 | 0.02 | 0.98 | 0.03 | 0.26 | 0.02 | 0.00 |
| 187 | *YML051W* | *GAL80* | 1.25 | 0.08 | 0.98 | 0.05 | 0.26 | 0.08 | 0.00 |
| 188 | *YHR096C* | *HXT5* | 1.32 | 0.04 | 1.06 | 0.01 | 0.27 | 0.04 | 0.00 |
| 189 | *YMR223W* | *UBP8* | 1.27 | 0.00 | 1.00 | 0.02 | 0.27 | 0.00 | 0.00 |
| 190 | *YGR200C* | *ELP2* | 1.04 | 0.00 | 0.76 | 0.03 | 0.27 | 0.00 | 0.00 |
| 191 | *YGL250W* | *RMR1* | 1.27 | 0.03 | 1.00 | 0.00 | 0.27 | 0.03 | 0.00 |
| 192 | *YJL169W* | *YJL169W* | 1.12 | 0.06 | 0.85 | 0.03 | 0.27 | 0.06 | 0.00 |
| 193 | *YDR517W* | *GRH1* | 1.22 | 0.01 | 0.95 | 0.02 | 0.27 | 0.01 | 0.00 |
| 194 | *YJR108W* | *ABM1* | 1.22 | 0.06 | 0.95 | 0.01 | 0.27 | 0.06 | 0.00 |
| 195 | *YPR132W* | *RPS23B* | 1.16 | 0.03 | 0.90 | 0.02 | 0.27 | 0.03 | 0.00 |
| 196 | *YDR374C* | *YDR374C* | 1.03 | 0.01 | 0.76 | 0.02 | 0.27 | 0.01 | 0.00 |
| 197 | *YKL026C* | *GPX1* | 1.14 | 0.05 | 0.86 | 0.01 | 0.27 | 0.05 | 0.00 |
| 198 | *YNL319W* | *YNL319W* | 1.07 | 0.07 | 0.80 | 0.03 | 0.28 | 0.07 | 0.00 |
| 199 | *YBR189W* | *RPS9B* | 1.15 | 0.01 | 0.88 | 0.02 | 0.28 | 0.01 | 0.00 |
| 200 | *YPL181W* | *CTI6* | 1.27 | 0.05 | 0.99 | 0.03 | 0.28 | 0.05 | 0.00 |
| 201 | *YIL077C* | *YIL077C* | 1.18 | 0.04 | 0.89 | 0.00 | 0.28 | 0.04 | 0.00 |
| 202 | *YKL179C* | *COY1* | 1.24 | 0.01 | 0.96 | 0.03 | 0.28 | 0.01 | 0.00 |
| 203 | *YNR064C* | *YNR064C* | 1.22 | 0.09 | 0.95 | 0.04 | 0.28 | 0.09 | 0.00 |
| 204 | *YPR151C* | *SUE1* | 1.20 | 0.04 | 0.92 | 0.02 | 0.29 | 0.04 | 0.00 |
| 205 | *YGL181W* | *GTS1* | 1.20 | 0.11 | 0.91 | 0.09 | 0.29 | 0.11 | 0.00 |
| 206 | *YOL053W* | *AIM39* | 1.02 | 0.05 | 0.73 | 0.05 | 0.29 | 0.05 | 0.00 |
| 207 | *YMR315W* | *YMR315W* | 1.20 | 0.04 | 0.90 | 0.01 | 0.29 | 0.04 | 0.00 |
| 208 | *YLR264W* | *RPS28B* | 1.15 | 0.03 | 0.86 | 0.02 | 0.29 | 0.03 | 0.00 |
| 209 | *YGL244W* | *RTF1* | 0.93 | 0.02 | 0.63 | 0.03 | 0.29 | 0.02 | 0.00 |
| 210 | *YMR100W* | *MUB1* | 1.15 | 0.03 | 0.87 | 0.04 | 0.30 | 0.03 | 0.00 |

**Table S1. SGAtool analyzed the scores of each knockout strain under HMF conditions (Continue)**

| NO | Array ORF | Array Name | Normalized colony size (EXPERIMENT) | Normalized colony std. dev. (EXPERIMENT) | Normalized colony size (CONTROL) | Normalized colony std. dev. (CONTROL) | Score | Score stdev | p-Value |
| --- | --- | --- | --- | --- | --- | --- | --- | --- | --- |
| 211 | *YJR011C* | *YJR011C* | 1.03 | 0.01 | 0.74 | 0.01 | 0.30 | 0.01 | 0.00 |
| 212 | *YLR172C* | *DPH5* | 1.09 | 0.06 | 0.79 | 0.00 | 0.30 | 0.06 | 0.00 |
| 213 | *YML102W* | *CAC2* | 1.27 | 0.02 | 0.97 | 0.01 | 0.30 | 0.02 | 0.00 |
| 214 | *YGR239C* | *PEX21* | 1.17 | 0.05 | 0.87 | 0.02 | 0.30 | 0.05 | 0.00 |
| 215 | *YOL007C* | *CSI2* | 1.26 | 0.00 | 0.96 | 0.02 | 0.30 | 0.00 | 0.00 |
| 216 | *YKR074W* | *AIM29* | 1.28 | 0.02 | 0.97 | 0.02 | 0.30 | 0.02 | 0.00 |
| 217 | *YMR219W* | *ESC1* | 1.32 | 0.05 | 1.02 | 0.02 | 0.30 | 0.05 | 0.00 |
| 218 | *YDR458C* | *HEH2* | 1.22 | 0.08 | 0.91 | 0.01 | 0.30 | 0.08 | 0.00 |
| 219 | *YOR139C* | *YOR139C* | 1.28 | 0.00 | 0.97 | 0.00 | 0.30 | 0.00 | 0.00 |
| 220 | *YHR111W* | *UBA4* | 1.21 | 0.02 | 0.90 | 0.02 | 0.31 | 0.02 | 0.00 |
| 221 | *YBR275C* | *RIF1* | 1.38 | 0.00 | 1.07 | 0.02 | 0.31 | 0.00 | 0.00 |
| 222 | *YDL093W* | *PMT5* | 1.18 | 0.02 | 0.87 | 0.02 | 0.31 | 0.02 | 0.00 |
| 223 | *YER067C-A* | *YER067C-A* | 1.18 | 0.05 | 0.86 | 0.01 | 0.32 | 0.05 | 0.00 |
| 224 | *YOL059W* | *GPD2* | 1.23 | 0.02 | 0.92 | 0.04 | 0.32 | 0.02 | 0.00 |
| 225 | *YKR103W* | *NFT1* | 1.32 | 0.03 | 1.00 | 0.00 | 0.32 | 0.03 | 0.00 |
| 226 | *YIL153W* | *RRD1* | 1.28 | 0.04 | 0.96 | 0.02 | 0.32 | 0.04 | 0.00 |
| 227 | *YOL080C* | *REX4* | 1.33 | 0.01 | 1.01 | 0.01 | 0.32 | 0.01 | 0.00 |
| 228 | *YDL187C* | *YDL187C* | 1.28 | 0.03 | 0.96 | 0.02 | 0.32 | 0.03 | 0.00 |
| 229 | *YLR004C* | *THI73* | 1.23 | 0.03 | 0.91 | 0.02 | 0.32 | 0.03 | 0.00 |
| 230 | *YHR034C* | *PIH1* | 1.18 | 0.04 | 0.85 | 0.01 | 0.33 | 0.04 | 0.00 |
| 231 | *YLR412W* | *BER1* | 1.14 | 0.01 | 0.82 | 0.01 | 0.33 | 0.01 | 0.00 |
| 232 | *YBR009C* | *HHF1* | 1.25 | 0.08 | 0.92 | 0.03 | 0.33 | 0.08 | 0.00 |
| 233 | *YJR058C* | *APS2* | 1.20 | 0.01 | 0.87 | 0.01 | 0.33 | 0.01 | 0.00 |
| 234 | *YLR131C* | *ACE2* | 1.27 | 0.03 | 0.93 | 0.00 | 0.33 | 0.03 | 0.00 |
| 235 | *YOR301W* | *RAX1* | 1.20 | 0.02 | 0.86 | 0.01 | 0.34 | 0.02 | 0.00 |
| 236 | *YNL206C* | *RTT106* | 1.19 | 0.01 | 0.86 | 0.01 | 0.34 | 0.01 | 0.00 |
| 237 | *YDL083C* | *RPS16B* | 1.19 | 0.03 | 0.85 | 0.02 | 0.34 | 0.03 | 0.00 |
| 238 | *YNL045W* | *LAP2* | 1.20 | 0.03 | 0.86 | 0.00 | 0.34 | 0.03 | 0.00 |
| 239 | *YDR313C* | *PIB1* | 1.30 | 0.01 | 0.95 | 0.01 | 0.35 | 0.01 | 0.00 |
| 240 | *YBR095C* | *RXT2* | 1.31 | 0.01 | 0.97 | 0.01 | 0.35 | 0.01 | 0.00 |
| 241 | *YJR087W* | *YJR087W* | 1.38 | 0.12 | 1.03 | 0.03 | 0.35 | 0.12 | 0.00 |
| 242 | *YDR257C* | *RKM4* | 1.34 | 0.06 | 0.98 | 0.04 | 0.35 | 0.06 | 0.00 |
| 243 | *YDL129W* | *YDL129W* | 1.44 | 0.03 | 1.08 | 0.01 | 0.36 | 0.03 | 0.00 |
| 244 | *YOR089C* | *VPS21* | 1.29 | 0.02 | 0.93 | 0.01 | 0.36 | 0.02 | 0.00 |
| 245 | *YOR135C* | *YOR135C* | 0.97 | 0.04 | 0.61 | 0.02 | 0.36 | 0.04 | 0.00 |

**Table S1. SGAtool analyzed the scores of each knockout strain under HMF conditions (Continue)**

| NO | Array ORF | Array Name | Normalized colony size (EXPERIMENT) | Normalized colony std. dev. (EXPERIMENT) | Normalized colony size (CONTROL) | Normalized colony std. dev. (CONTROL) | Score | Score stdev | p-Value |
| --- | --- | --- | --- | --- | --- | --- | --- | --- | --- |
| 246 | *YLR423C* | *ATG17* | 1.29 | 0.01 | 0.92 | 0.05 | 0.36 | 0.01 | 0.00 |
| 247 | *YOR045W* | *TOM6* | 1.30 | 0.02 | 0.94 | 0.01 | 0.36 | 0.02 | 0.00 |
| 248 | *YBR082C* | *UBC4* | 1.29 | 0.03 | 0.91 | 0.03 | 0.37 | 0.03 | 0.00 |
| 249 | *YKL213C* | *DOA1* | 1.26 | 0.03 | 0.89 | 0.01 | 0.37 | 0.03 | 0.00 |
| 250 | *YLR418C* | *CDC73* | 0.92 | 0.02 | 0.55 | 0.02 | 0.37 | 0.02 | 0.00 |
| 251 | *YPL032C* | *SVL3* | 1.16 | 0.03 | 0.79 | 0.03 | 0.37 | 0.03 | 0.00 |
| 252 | *YPL139C* | *UME1* | 1.28 | 0.03 | 0.91 | 0.01 | 0.37 | 0.03 | 0.00 |
| 253 | *YMR252C* | *YMR252C* | 1.21 | 0.03 | 0.83 | 0.01 | 0.37 | 0.03 | 0.00 |
| 254 | *YLR048W* | *RPS0B* | 1.15 | 0.03 | 0.77 | 0.01 | 0.38 | 0.03 | 0.00 |
| 255 | *YMR124W* | *YMR124W* | 1.20 | 0.00 | 0.82 | 0.02 | 0.38 | 0.00 | 0.00 |
| 256 | *YMR274C* | *RCE1* | 1.29 | 0.05 | 0.91 | 0.01 | 0.38 | 0.05 | 0.00 |
| 257 | *YLR174W* | *IDP2* | 1.34 | 0.05 | 0.94 | 0.03 | 0.39 | 0.05 | 0.00 |
| 258 | *YLR388W* | *RPS29A* | 1.20 | 0.03 | 0.81 | 0.00 | 0.39 | 0.03 | 0.00 |
| 259 | *YAR002W* | *NUP60* | 1.32 | 0.01 | 0.93 | 0.01 | 0.39 | 0.01 | 0.00 |
| 260 | *YHR048W* | *YHK8* | 1.33 | 0.05 | 0.93 | 0.04 | 0.40 | 0.05 | 0.00 |
| 261 | *YOR007C* | *SGT2* | 1.14 | 0.03 | 0.73 | 0.00 | 0.40 | 0.03 | 0.00 |
| 262 | *YMR073C* | *IRC21* | 1.27 | 0.01 | 0.87 | 0.01 | 0.41 | 0.01 | 0.00 |
| 263 | *YMR025W* | *CSI1* | 1.44 | 0.09 | 1.02 | 0.03 | 0.41 | 0.09 | 0.00 |
| 264 | *YOL103W* | *ITR2* | 1.17 | 0.05 | 0.75 | 0.02 | 0.42 | 0.05 | 0.00 |
| 265 | *YIL008W* | *URM1* | 1.31 | 0.06 | 0.85 | 0.04 | 0.45 | 0.06 | 0.00 |
| 266 | *YML048W-A* | *YML048W-A* | 1.24 | 0.04 | 0.79 | 0.03 | 0.45 | 0.04 | 0.00 |
| 267 | *YML010C-B* | *YML010C-B* | 1.37 | 0.08 | 0.92 | 0.01 | 0.46 | 0.08 | 0.00 |
| 268 | *YKL066W* | *YKL066W* | 1.47 | 0.01 | 1.00 | 0.02 | 0.47 | 0.01 | 0.00 |
| 269 | *YKR072C* | *SIS2* | 1.43 | 0.04 | 0.97 | 0.01 | 0.47 | 0.04 | 0.00 |
| 270 | *YLR218C* | *COA4* | 1.35 | 0.06 | 0.88 | 0.02 | 0.48 | 0.06 | 0.00 |
| 271 | *YOR312C* | *RPL20B* | 1.46 | 0.11 | 0.97 | 0.01 | 0.48 | 0.11 | 0.00 |
| 272 | *YML102C-A* | *YML102C-A* | 1.31 | 0.02 | 0.83 | 0.01 | 0.48 | 0.02 | 0.00 |
| 273 | *YMR105C* | *PGM2* | 1.45 | 0.01 | 0.96 | 0.03 | 0.49 | 0.01 | 0.00 |
| 274 | *YOL068C* | *HST1* | 1.35 | 0.02 | 0.87 | 0.03 | 0.49 | 0.02 | 0.00 |
| 275 | *YKL037W* | *AIM26* | 1.12 | 0.02 | 0.62 | 0.00 | 0.50 | 0.02 | 0.00 |
| 276 | *YHR003C* | *YHR003C* | 1.42 | 0.02 | 0.92 | 0.01 | 0.50 | 0.02 | 0.00 |
| 277 | *YKL074C* | *MUD2* | 1.41 | 0.01 | 0.90 | 0.03 | 0.50 | 0.01 | 0.00 |
| 278 | *YIL108W* | *YIL108W* | 1.41 | 0.05 | 0.91 | 0.04 | 0.50 | 0.05 | 0.00 |
| 279 | *YCR063W* | *BUD31* | 1.26 | 0.01 | 0.75 | 0.03 | 0.51 | 0.01 | 0.00 |
| 280 | *YGR201C* | *YGR201C* | 1.55 | 0.04 | 1.04 | 0.03 | 0.51 | 0.04 | 0.00 |

**Table S1. SGAtool analyzed the scores of each knockout strain under HMF conditions (Continue)**

| NO | Array ORF | Array Name | Normalized colony size (EXPERIMENT) | Normalized colony std. dev. (EXPERIMENT) | Normalized colony size (CONTROL) | Normalized colony std. dev. (CONTROL) | Score | Score stdev | p-Value |
| --- | --- | --- | --- | --- | --- | --- | --- | --- | --- |
| 281 | *YGR170W* | *PSD2* | 1.29 | 0.04 | 0.77 | 0.05 | 0.53 | 0.04 | 0.00 |
| 282 | *YCL037C* | *SRO9* | 1.37 | 0.01 | 0.82 | 0.01 | 0.55 | 0.01 | 0.00 |
| 283 | *YAL013W* | *DEP1* | 1.36 | 0.09 | 0.80 | 0.02 | 0.57 | 0.09 | 0.00 |
| 284 | *YDR348C* | *PAL1* | 1.55 | 0.02 | 0.98 | 0.01 | 0.57 | 0.02 | 0.00 |
| 285 | *YGR202C* | *PCT1* | 1.51 | 0.04 | 0.94 | 0.03 | 0.58 | 0.04 | 0.00 |
| 286 | *YNL040W* | *YNL040W* | 1.58 | 0.05 | 1.00 | 0.03 | 0.59 | 0.05 | 0.00 |
| 287 | *YPL182C* | *YPL182C* | 1.61 | 0.07 | 1.01 | 0.02 | 0.59 | 0.07 | 0.00 |
| 288 | *YJL016W* | *YJL016W* | 1.44 | 0.04 | 0.84 | 0.02 | 0.60 | 0.04 | 0.00 |
| 289 | *YKL015W* | *PUT3* | 1.58 | 0.15 | 0.95 | 0.01 | 0.63 | 0.15 | 0.00 |
| 290 | *YKL076C* | *YKL076C* | 1.54 | 0.06 | 0.90 | 0.01 | 0.64 | 0.06 | 0.00 |
| 291 | *YOL004W* | *SIN3* | 1.39 | 0.02 | 0.73 | 0.01 | 0.66 | 0.02 | 0.00 |
| 292 | *YKL069W* | *YKL069W* | 1.36 | 0.02 | 0.70 | 0.01 | 0.66 | 0.02 | 0.00 |
| 293 | *YKR082W* | *NUP133* | 1.37 | 0.02 | 0.68 | 0.02 | 0.68 | 0.02 | 0.00 |
| 294 | *YMR106C* | *YKU80* | 1.69 | 0.00 | 0.96 | 0.02 | 0.74 | 0.00 | 0.00 |

**Figure S1.** **Growth curves of BY4741 and** ***Siw14Δ* in YPD+G418 liquid medium**

**A**

**
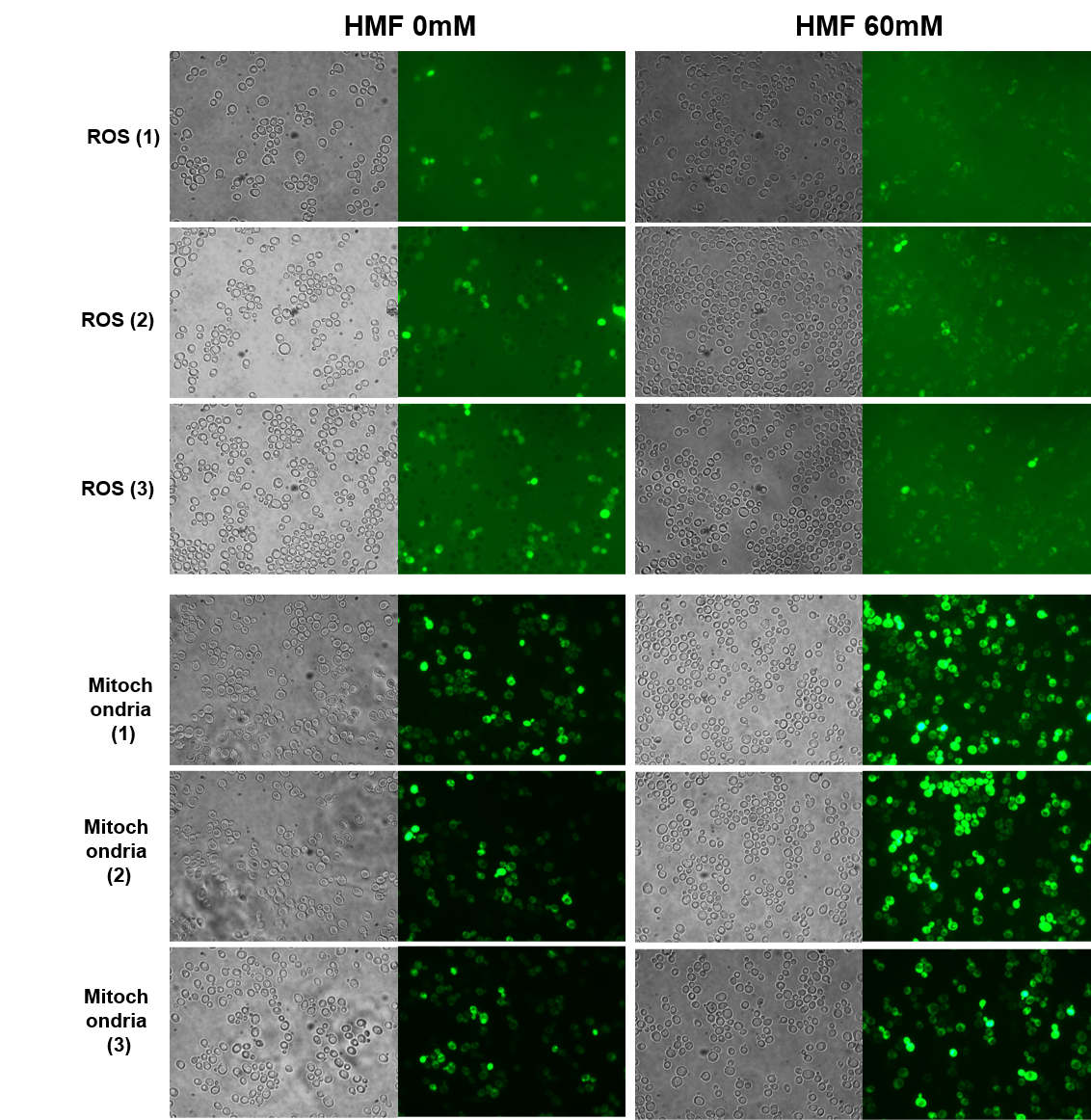
**

**B**

**
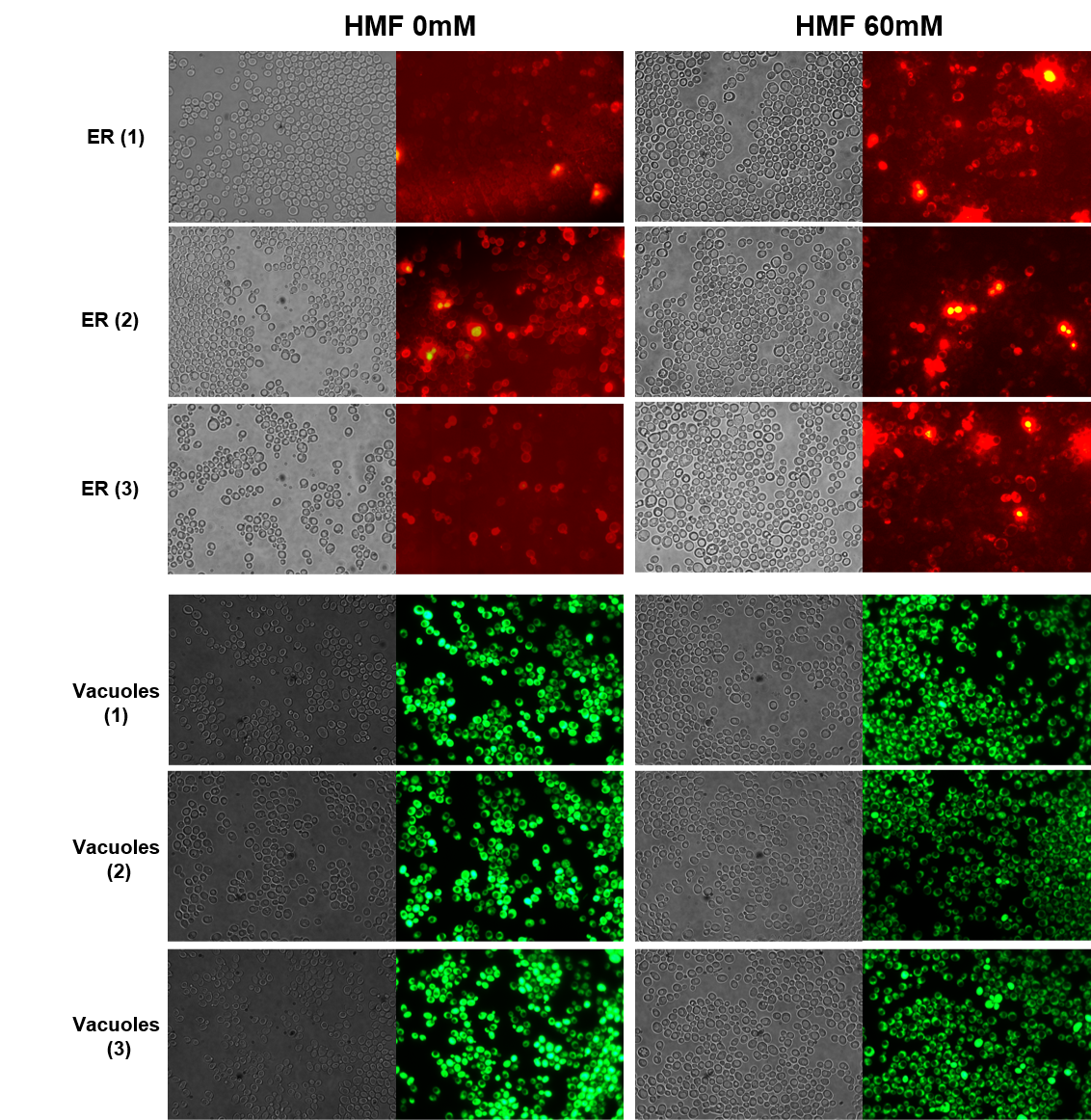
**

**C**

**
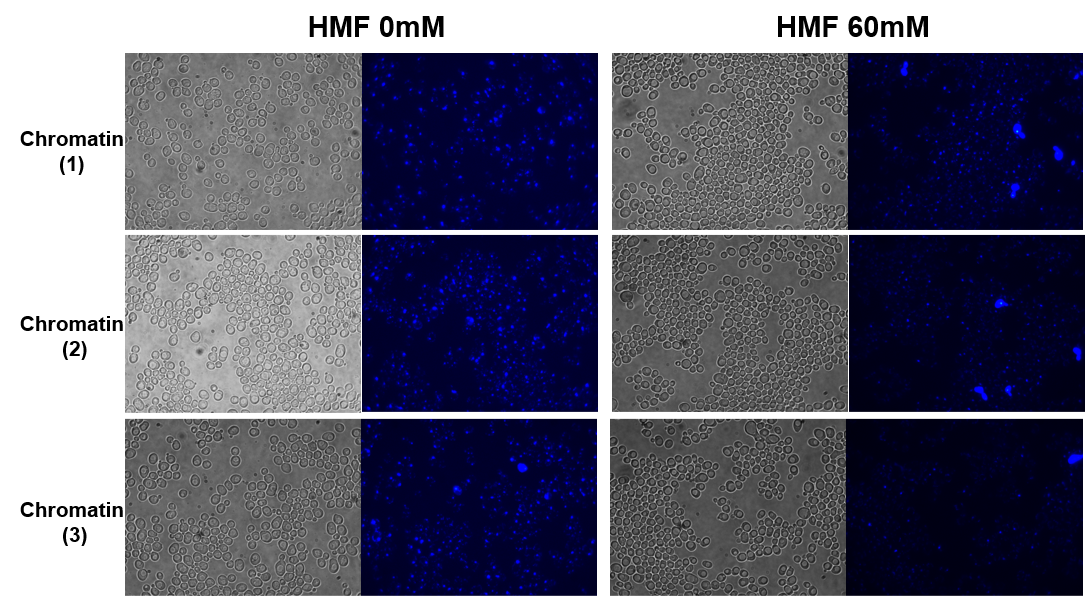
**

**Figure S2.** **The original image from the subcellular observation experiment with BY4741**

(**A**) Reactive Oxygen species and Mitochondria. (**B**) Endoplasmic reticulum and Vacuoles. (**C**) Chromatin.

**A**

**
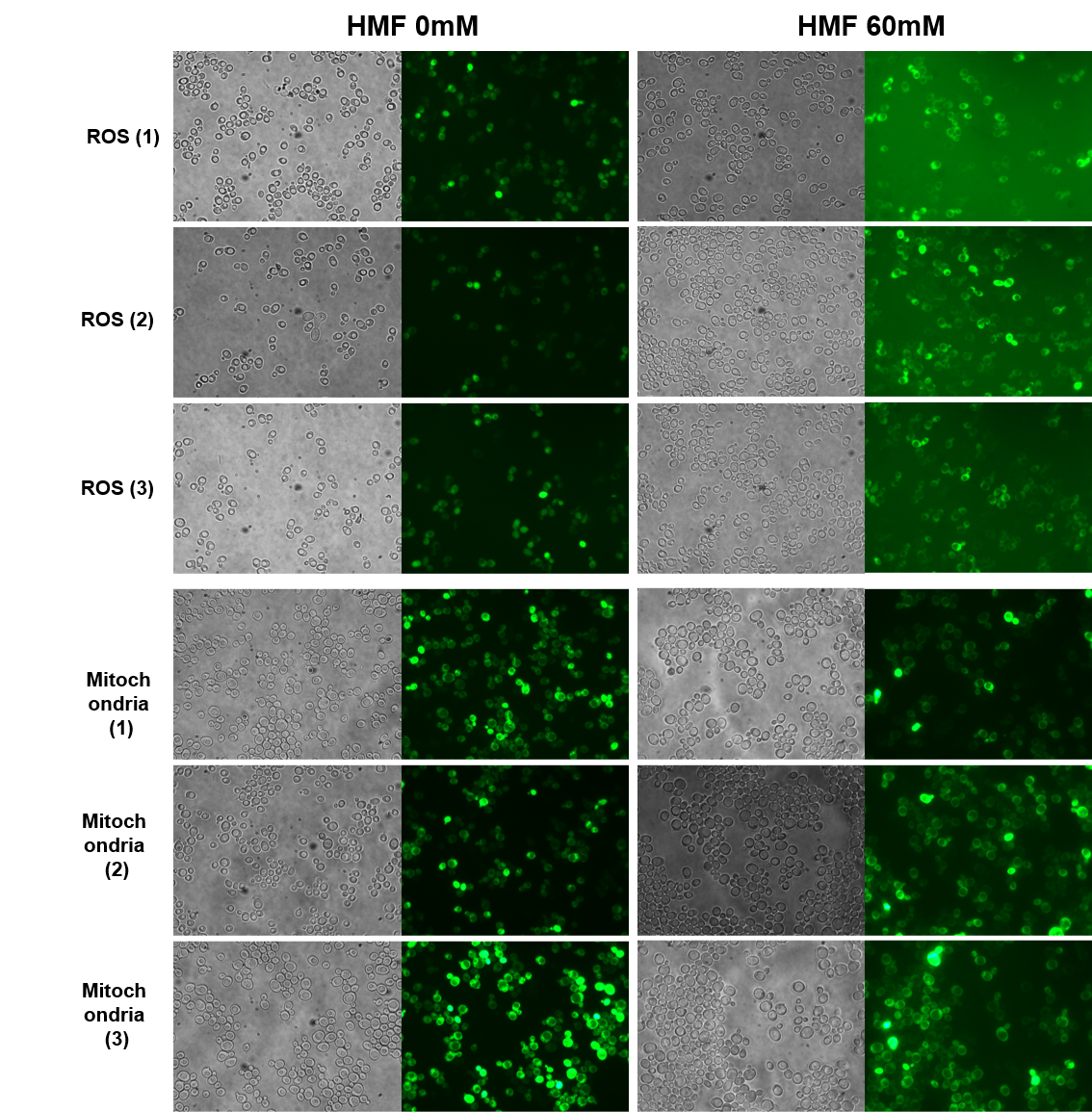
**

**B**

**
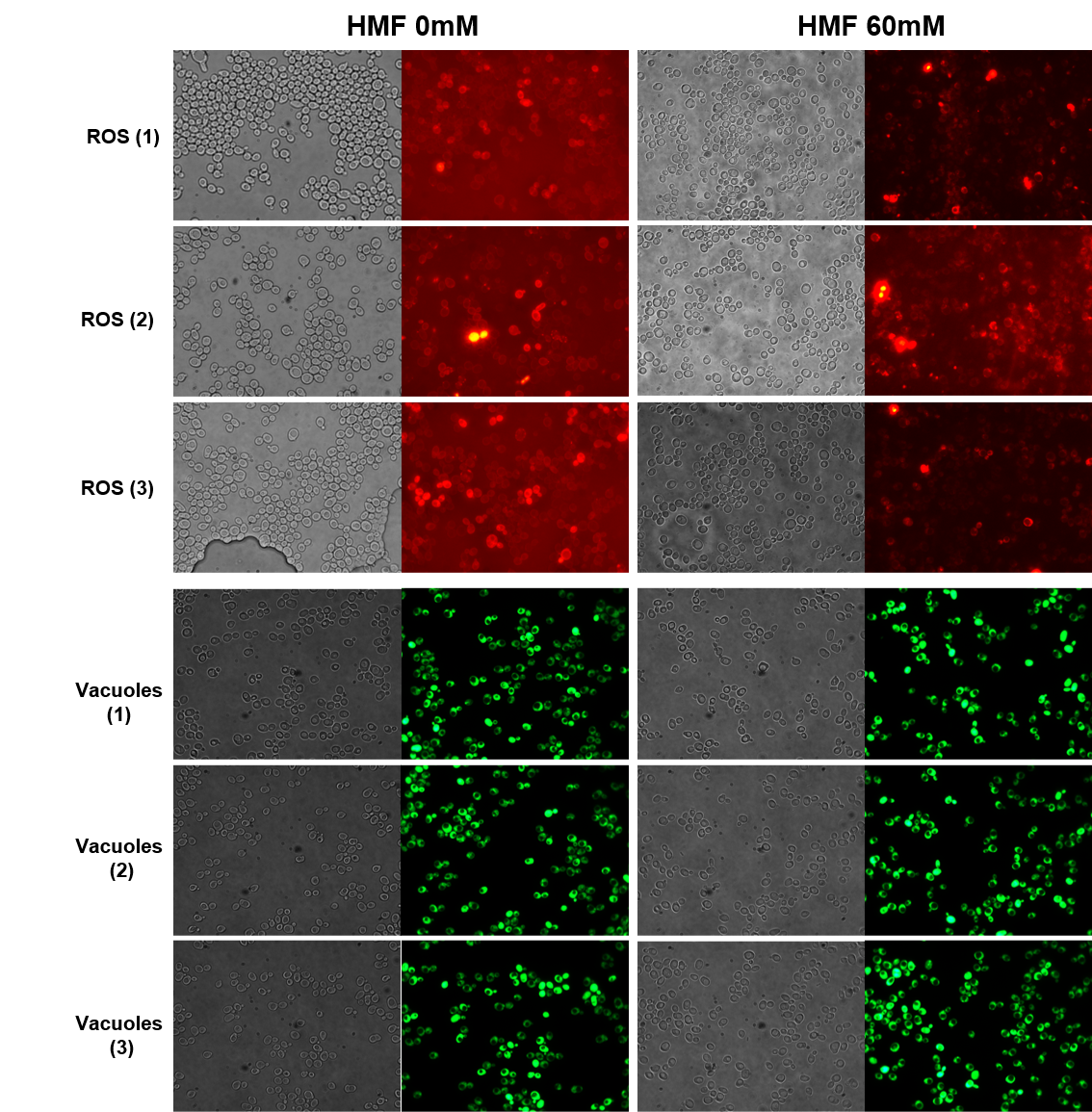
**

**C**

**
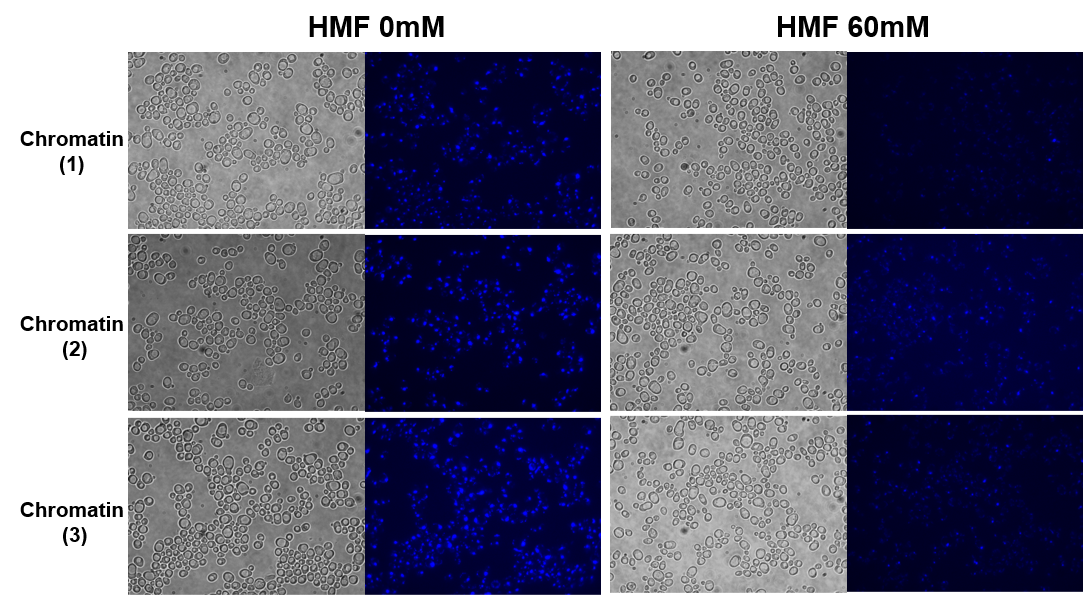
 Figure S3.** **The original image from the subcellular observation experiment with Siw14Δ**

(**A**) Reactive Oxygen species and Mitochondria. (**B**) Endoplasmic reticulum and Vacuoles. (**C**) Chromatin.
